# Recent Advances in Gadolinium Based Contrast Agents for Bioimaging Applications

**DOI:** 10.3390/nano11092449

**Published:** 2021-09-20

**Authors:** Atiya Fatima, Md. Wasi Ahmad, Abdullah Khamis Ali Al Saidi, Arup Choudhury, Yongmin Chang, Gang Ho Lee

**Affiliations:** 1 Department of Chemical Engineering, College of Engineering, Dhofar University, P.O. Box 2509, Salalah 211, Sultanate of Oman; atiyaqazi@gmail.com; 2 Department of Chemistry, College of Natural Sciences, Kyungpook National University (KNU), Taegu 702-701, Korea; abdullah_al_saidi@hotmail.com; 3 Department of Chemical Engineering, Birla Institute of Technology, Ranchi 835215, India; 4 Department of Molecular Medicine and Medical & Biological Engineering, School of Medicine, Kyungpook National University (KNU), Taegu 702-701, Korea; ychang@knu.ac.kr

**Keywords:** gadolinium based contrast agents, nanoparticles, gadolinium oxide nanoparticles, coating ligands, magnetic resonance imaging, bio-imaging applications

## Abstract

Gadolinium (Gd) based contrast agents (CAs) (Gd-CAs) represent one of the most advanced developments in the application of Gd for magnetic resonance imaging (MRI). Current challenges with existing CAs generated an urgent requirement to develop multimodal CAs with good biocompatibility, low toxicity, and prolonged circulation time. This review discussed the Gd-CAs used in bioimaging applications, addressing their advantages and limitations. Future research is required to establish the safety, efficacy and theragnostic capabilities of Gd-CAs. Nevertheless, these Gd-CAs offer extraordinary potential as imaging CAs and promise to benefit bioimaging applications significantly.

## 1. Introduction

Nanotechnology has paved the way for the development of new therapeutics leading to efficient theragnostics of severe diseases. In the last few decades, the integration of nanoparticles (NPs) has dramatically impacted medical imaging. Significant advances have evolved in synthetic methodologies to develop a variety of nanomaterials with controllable size and shape, physicochemical characteristics, and surface charge of nanomaterials. These nanomaterials could be decorated with other NPs, polymers, and bioactive compounds to enhance their targeting, biosensing, and biocompatibility. These technological advances have exemplified their importance in biological imaging applications [1]. The utilization of NPs in disease diagnosis dates back to 1990, when it was first used commercially as a liver imaging agent and is currently used for bowel imaging under the trade names Gastromarks/Lumirems [2]. Subsequent progress in understanding size-dependent physical and chemical parameters of NPs has led to substantial progress in this research area. Many non-invasive medical imaging methodologies have been developed and are used in clinical diagnosis and drug discovery research. These molecular imaging techniques involve advancement in molecular probes to visualize cellular function and characterize and measure the molecular processes inside the living organisms [3]. The evolution in synthetic technologies of NPs in collaboration with the intense development of imaging modalities has significantly advanced disease detection. Progress in targeted contrast agents (CAs) has advanced the selective imaging of specific biological events and processes considerably with enhanced detection limits and imaging modalities, establishing these CAs as a mainstay in current medicinal and biological research. NPs passively target tumors via enhanced permeability and retention effect (EPR) without the help of any exogenous targeting ligands [4] and can target specific tissues through molecular sieving [5]. Conjugated NPs-tumor-targeting ligands (peptides, antibodies, etc.) can successfully target specific tumors with high precision [6].

This review emphasizes recent advances in the development of molecular imaging probes using Gadolinium-based contrast agents (Gd-CAs) with a particular focus on their bioimaging applications.

## 2. Bioimaging Techniques

Several non-invasive imaging techniques such as optical imaging (OI), magnetic resonance imaging (MRI), ultrasound (US), computed tomography (CT), positron emission tomography (PET), and single-photon emission computed tomography (SPECT) have been developed. These techniques differ in the context of their resolution, sensitivity, complexity, time of data acquisition and cost-effectiveness. Therefore, these techniques are often used as complementary techniques with a choice of selected imaging modality depending upon the type of requirement. A brief description of these techniques is summarized in Table 1. 

### 2.1. Magnetic Resonance Imaging (MRI)

The MRI technique operates on the basic nuclear magnetic resonance (NMR) principles and generates images using the tissue contrast produced through NMR signals. Gadolinium-based MRI contrast agents operate by shortening the T_1_ and T_2_ relaxation time of protons present inside the tissues, thus enhance the image contrast. This contrast imaging can be further improved by the use of CAs, which enhances the signal intensity between the tissue of interest (tumor) and the background tissue (normal tissues). There are several parameters such as longitudinal relaxation time (T_1_), Transverse relaxation time (T_2_), and Spin density (ρ) which affect the signal intensity and the extent of contrast obtained from a sample. These aspects are crucial in designing MRI contrast agents, which aids in generating contrast images for diagnosis. MRIs are limited by the factors of cost and longer imaging times. Nevertheless, MRI contrast agents can efficiently detect lesions and differentiate them from healthy tissues [9]. 

### 2.2. Optical Imaging (OI)

Optical imaging utilizes different physical parameters of light interaction with tissues to generate images. Among all the optical imaging techniques, fluorescence microscopy has evolved as a prominent imaging technique that depends on the fluorophore’s inherent property (i.e., lanthanide compounds, organic dyes, etc.). Fluorescence imaging (FI), and particularly near-infrared fluorescence (NIRF) imaging, provides the highest spatial resolution on a microscopic level for disease diagnosis. However, this technique is limited by the factors of limited penetration depth and auto-fluorescence in various tissues, which hinders its clinical utility [10]. Several efforts have been made to overcome these limitations by the use of NPs. Surplus loading of fluorescent dyes in NPs can increase signal [11], modified NPs can prevent the NIRF quenching [12], and local lesion concentration of the fluorescent dye can be increased by increasing NPs concentration [13]. There have been several studies where nanoparticle FI was used to detect genes, analyze protein, evaluate enzyme activity, trace elements, and cells and for the early-stage diagnosis of tumors [14,15,16,17,18]. 

### 2.3. Computed Tomography (CT) Imaging

A CT scan generates tomographic (cross-sectional) images of a tissue using X-ray measurements taken from different angles. Although this technique involves larger radiation exposure, it still has gained importance due to its faster examination speed, improved efficiency, high spatial resolution, and cost-effectiveness. Current CT contrast agents are mainly iodine-based and limited by fast clearance rates and adverse side effects. Nanosized CT contrast agents have emerged as potential substitutes surmounting these limitations. There are two methods to synthesize these contrast agents; the first is iodine-based contrast agents where iodine is loaded with NPs [19], and the second is based on metals NPs having high X-ray attenuation coefficients [20]. These contrast agents based on metal NPs are utilized in multiple areas based on their ability to generate attenuation, cellular uptake and targeting capabilities. 

### 2.4. Ultrasound (US) Imaging

Ultrasound (US) imaging is a technique that utilizes pulses of US to generate images of tissue using a probe where the US pulses are echoed off tissues with different reflection properties returning back to the probe, which records and exhibit them as an image. It is one of the most commonly used imaging techniques owing to its high spatial resolution, probability, and cost-effectiveness. Commercial US contrast agents are comprised of microbubbles [21]. These microbubbles are limited by the factors of relatively short circulation lifetime, low stability and provide only blood pool contrast signals [22]. NPs with particle sizes ranging from 100–1000 nm have been investigated to surmount these limitations [23]. These NPs can be attached with several surface ligands to impart specific target binding abilities. These US contrast agents are synthesized in three types based on their composition. Most common agents utilize microbubbles (gas-based) to create acoustic reflections. The second type is smaller than gas-based particles and utilizes a solid-based NP with relatively larger scattering acoustic signals. The third type is based on liquid-based NP, which generates an acoustic signal due to the difference in speeds of sound transmission and water. These NP contrast agents are comparatively much smaller than current US contrast agents used and, due to their smaller size and supplementary surface labelling, provide better lesion targeting.

### 2.5. Positron Emission Tomography (PET), Single Photon Emission Computed Tomography (SPECT) Imaging

PET and SPECT are imaging techniques that provide detailed metabolic information. PET provides real-time quantitative imaging analysis with high tissue penetration and high sensitivity. SPECT possesses similar advantages as PET imaging, but both are disadvantageous in high cost and radioactive exposure. In addition, PET/SPECT NPs imaging tracers require nuclides with a long half-life, where radionuclides used in SPECT generally have longer half-lives. They are most commonly used in tumor imaging, where the images are obtained via specific binding to receptors [24].

## 3. Nanoparticles in Molecular Imaging

In the last few decades, nanomaterials have gained considerable attention and have been extensively explored for their potential application in the field of molecular imaging, especially in cancer therapeutics [25]. Various parameters such as particles size, shape, charge, and hydrophilicity of the NPs are crucial for the effectiveness of the designed contrast agents. Medical imaging CAs currently used are mostly the small molecules exhibiting swift metabolism, having a non-specific distribution with undesirable toxicities [26]. NPs owing to their nano size, exhibit greater permeability and retention effects inside the tumor cells [27]. Nanoparticle’s size plays a crucial role in biodistribution, cellular uptake, blood circulation half-life and tumor penetration [28]. NPs having a particle size smaller than 10 nm pass through the renal filtration pore having a pore size of 10 nm and thus get rapid clearance through the renal excretion system [29,30], whereas NPs over 100 nm in size can be easily identified by macrophages and accumulate in lung, liver, lymph nodes and spleen [31], while other reports suggested that NPs with size range between 10 and 60 nm have increased cellular uptake [32]. Particle charge is yet another factor; positively charged particles improve endocytosis or phagocytosis for cell labelling [33]. Modification and functionalization of NPs provide a platform for improvement over current contrast media by improving specificity, prolonged circulation half-life, and in vitro and in vivo stability [30,34,35,36]. The addition of targeting ligands significantly enhances the specificity and increases nanoparticle–tumor interactions for tumorous tissues [37]. NPs functionalized with targeting ligands such as peptides, antibodies, small molecules and proteins for efficient drug delivery and diagnosis [38,39,40,41,42]. Functionalization of NPs have been reported to achieve higher affinity towards certain cell surface receptor proteins overexpressed in various cancer cells [43]. Another approach is modifying NPs using antibodies that impart labelling of cells and tissues but is limited to cost-effectiveness and smaller shelf life [3]. Small organic molecules are conjugated with NPs to impart bioimaging which is an excellent alternative to modifies NPs having limited shelf life [44,45]. Certain factors are crucial in the synthesis of NPs for bioimaging applications. The primary requirement is the synthesis of optical core encapsulating the fluorochrome [46,47,48,49]. To improve the photostability and for the protection of the optical core, a shell is synthesized. For the prevention of coagulation or agglomeration, it is necessary to modify the NPs to maintain a dispersed state which is achieved by using surfactants, polymers, chelating groups, etc. [50,51]. Finally, to achieve specificity and increase the bioconjugation and targeting of NPs, it is necessary to attach them to suitable biomolecules such as enzymes, antibodies, peptides, drugs etc., which also promotes or maintains their dispersion [40,52,53,54]. All of these factors are required in designing optimum nanoparticulate systems for bio-imaging applications.

Most of the clinically available CAs used in MRI are based on paramagnetic Gd complexes [55]. Magnevist, a Gadolinium (Gd) (III) chelate, is a widely used MRI Cas [56,57]. However, it is associated with potential biotoxicity issues. It is limited by the factors of relatively lower relaxivity and shorter blood circulation time [58,59,60]. Gadolinium (III)-based NPs (Gd-NPs) have found their place in biomedical applications due to their excellent characteristics. They are utilized to formulate enhanced CAs as these NPs are thermodynamically and kinetically stable, possess high relaxivity, have good water solubility and in vivo stability, exhibit low toxicity, and offer better control over the molecular size and functionalization [61,62,63,64,65,66,67]. Free Gadolinium (Gd) ions are toxic in nature and pose significant threats to the human body in their free form. To reduce the toxicity of free Gd ions, they are encapsulated by a ligand or inside a material. Two principal ways are utilized to exploit their properties as nanomaterials: (i) grafting of Gd chelates inside or on the surface of the nanomaterials, (ii) development of Gd crystalline NPs. These methods are advantageous in increasing the number of Gd in the CAs for obtaining a better signal or to increase the therapeutic effects [68]. The trivalent Gd(III) ion possesses the highest electron spin magnetic moment (S = 7/2) of elements in the periodic table as it has the greatest number of unpaired electrons (seven) in its 4f-orbital (^8^S_7/2_). Owing to this, Gd-NPs has gained significant attention due to their enhanced r_1_ values, owing to a large amount of Gd per nanoparticle, providing stronger contrast [69,70]. The r_1_ and transverse water proton spin relaxivity (r_2_) of Gd-NPs depend on the ligand size coated on its surface. Kim et al. studied this dependence and found that r1 and r2 values decreased with increasing ligand size due to the ligand size effect [71]. In another study, the relaxometric properties of Gd-NPs coated with several ligands such as small diacids with hydrophobic chains, namely, succinic acid, glutaric acid, and terephthalic acid, and large polyethyleneimine (PEI) with hydrophilic chains, namely, PEI-1300 and PEI-10000 were investigated, where ligand-size and ligand-chain hydrophilicity effects were observed. The r_1_ and r_2_ water proton relaxivities were found to be decreasing with increasing ligand-size. The ligand-size effect was weaker in case of PEI since its hydrophilic chains permitted water molecules to access the NPs (the ligand-chain hydrophilicity effect). This result was explained on the basis of the magnetic dipole interaction between the dipoles of the nanoparticle and water protons [72]. The r_2_/r_1_ ratio of T_1_ MRI contrast agent is also an important factor while designing contrast agents. An ideal T_1_ MRI contrast agent should have a r_2_/r_1_ ratio close to 1. Bony et al. synthesized d-glucuronic acid-coated Cu(II)/Gd(III) oxide nanoparticle, which showed r_1_ = 13.78 s^−1^·mM^−1^ and r_2_ = 14.48 ^ ^s^−1^·mM^−1^ (r_2_/r_1_ = 1.05). This is due to the reduction in magnetization of mixed NPs caused by the mixing of NPs [73].

**Figure 1:**
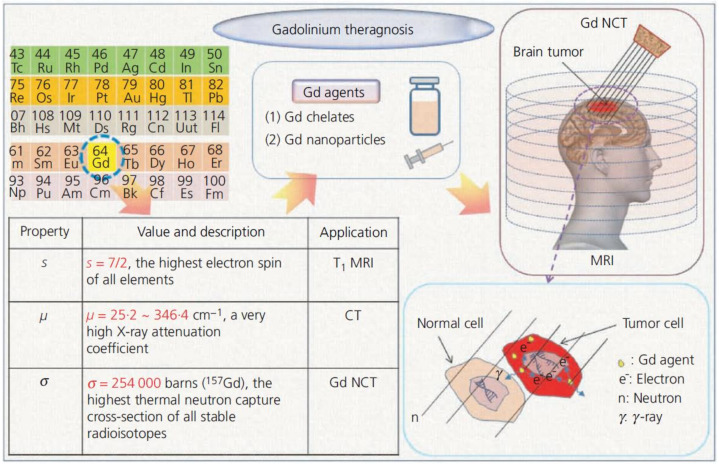
Outline of Gd theragnosis of malignant tumors (a brain tumor is illustrated). Reprinted with permission from [74]. Copyright 2012 Thomas Telford, Ltd.

The properties of Gd include the largest spin magnetic moment among all the elements, a high X-ray attenuation coefficient (m) and the highest thermal neutron capture cross-section (s) among all stable radioisotopes. The first property allows Gd agents to have a very high longitudinal water proton relaxivity (r_1_), useful for positive (T_1_) MRI. The second property is effective for CT. The third property is beneficial for neutron capture therapy (NCT) of tumors. Given these properties, Gd agents might be used for the theragnosis a malignant tumor (i.e., diagnose the malignant tumor by way of T_1_ MRI or CT and treat it by NCT) as outlined in Figure 1 [74]. Table 2 represent the Single/multimodal bioimaging application of Gd-CAs. 

## 4. Functionalization of Gd-NPs

Gd-NPs have attractive characteristics for the development of CAs, and the exploitation of these features were feasible after an effective synthesis of Gd-NPs, and their functionalization was developed. Gd-NPs have been conjugated with innumerable compounds, other NPs and chelating agents to offer efficient pharmacokinetic characteristics and better control over biodistribution. There are several examples such as, Gd-NPs-diethylenetriamine pentaacetic acid (Gd-NPs-DTPA) or Gd-NPs-DOTA functionalized polymer [85,86], self-assembled peptide amphiphiles [87,88] or viral capsid [89], Gd-NPs-DTPA terminated dendrimer [90,91], Gd complexes loaded liposomes [92], high-density lipoprotein NPs [93], micelles [94] or polymeric NPs [95], Gd ions entrapped in zeolites [96], fullerenes [97], carbon nanotubes [98] clays [99] or mesoporous silica NPs (MSNPs) [100], and Gd chelates immobilized on quantum dots (QDs) [101], on lipid particles [102] and on Au-NPs [103] were synthesized and studied. Some of these are functionalized by fluorescent molecules or bio targeting groups which conferred additional attractive features. Apart from this, the potential of crystalline NPs based on Gd_2_O_3_ (Gd-NPs) [104,105,106], Gd fluoride NPs [107], and Gd carbonate [108,109] have also been evaluated. The synthesis and application of Gd-CAs conjugated with several natural and synthetic polymers, saccharides and various organic and inorganic compounds are discussed in this section.

### 4.1. Naturally Derived Polymers

**Figure 2:**
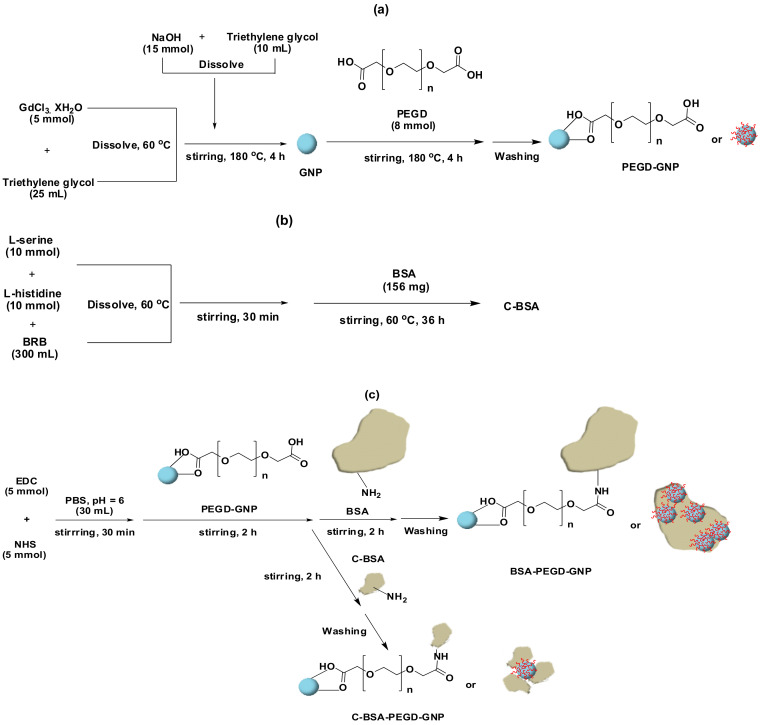
Synthesis of (**a**) Gd-NPs and PEGD-Gd-NPs, (**b**) the C-BSA, and (**c**) the BSA-PEGD-Gd-NPs and C-BSA-PEGD-GNPs. Reprinted with permission from [44]. Copyright 2014 Elsevier.

Functionalization of Gd compounds with naturally derived polymers such as proteins, liposomes, saccharides etc., is obtained through chemical conjugation, chelation or encapsulation. These polymers enhance relaxivity, provides additional functionalization sites and greatly increases the biocompatibility of the synthesized agents. Albumins are globular proteins, mostly serum albumins. They have been extensively utilized in developing Gd-CAs due to their amphiphilic character and affinity towards a variety of malignant tumors. In a study, Gd–albumin conjugates were synthesized having relaxivity of approximately 9~10.5 s^−1^·mM^−1^ at 3 tesla (T), which is greater than the reported value for Magnevist^®^ [110]. In another approach, encapsulation of Gd-NPs inside the albumin-folic acid NPs resulted in reduced toxicity and increased r1 value of 10.8 s^−1^ mM^−1^ at 0.47 T [111]. Ahmad et al. synthesized polyethylene glycol diacid (PEGD) coated Gd-NPs (PEGD-Gd-NPs, the average particle diameter (d_avg_ = 2.0 nm)), conjugated with bovine serum albumin (BSA/cleaved-BSA (C-BSA) (i.e., BSA-PEGD-Gd-NPs and C-BSA-PEGD-Gd-NPs) by reacting amine group of albumins with the acidic moiety of PEGD (Figure 2). Large relaxivities values i.e., r_1_ = 6.0 s^−1^·mM^−1^ and r_2_ = 28.0 s^−1^ mM^−1^ for BSA-PEGD-Gd-NPs and r_1_ = 7.6 s^−1^·mM^−1^ and r_2_ = 22.0 s^−1^·mM^−1^ for C-BSA-PEGD-Gd-NPs, were observed along with significant negative contrast enhancements [44].

Gd-NPs coated with trans-activator of transcription (TAT) peptide with the cell-penetrating ability (i.e., TAT-Gd-NPs) were synthesized through a one-pot process exhibiting r_1_ value greater than those of commercial Gd-chelates. Synthesized TAT-Gd-NPs possessed the d_avg_ of 1.5 nm with a r_1_ of 18.2 s^−1^·mM^−1^ and r_2_/r_1_ = 1.6 [112]. There are several cancer-related proteins that are abundantly present in the tumor extracellular matrix. These proteins can be used to functionalize Gd-CAs to impart tumor specificity. Therefore, cyclic RGD-conjugated Gd-NPs have been reported to be employed for tumor-targeting T_1_ MRI. Five types of commercial cyclic RGDs (cRGDs) were used as a tumor-targeting ligand to coat Gd-NPs having particle d_avg_ ranging from 1.0–2.5 nm. Synthesized conjugates exhibited r_1_ values of 10.0–18.7 s^−1^·mM^−1^, with r_2_/r_1_ ratios of 1.4–1.7, which is approximately 3–5 times higher than the values reported for commercial Gd chelates. In addition, approximately 3 times contrast enhancements in the T_1_ MR images were observed [25]. In a similar approach, Gd-DOTA-cRGD was used to target tumor correlated α_v_β_3_-receptor. Synthesized Gd-CAs exhibited relaxivity r_1_ of ~7.4 s^−1^·mM^−1^ at 1.5 T (64 MHz) and moderate specificity for the α_v_β_3_-receptor in hepatocellular carcinoma in in vivo studies [113]. Yang et al. developed a Gd-integrated polypyrrole nano-theragnostic agent (PPy@BSA-Gd) by using BSA as polymerization and biomimetic mineralization stabilizer, possessing high stability and appreciable photothermal property. These agents possess good cytocompatibility and a relaxivity value, r_1_ = 10.203 s^−1^·mM^−1^, posing them as a potential probe for T_1_ MRI and applications in photothermal therapy [114].

### 4.2. Saccharides and Their Derivatives

Saccharides stimulate the cell response and are widely used as tumor targeting agents [115]. Gd-CAs modified with several saccharides or their derivative have gained attention by researchers. For instance, cyclodextrin (CD), glucosamine, chitosan (CS), and dextran conjugation with Gd-CAs have been reported to impart tumor specificity and enhance biocompatibility. Gd-CAs modified with saccharides, and their derivatives have become a common approach, especially CD [116], dextran [117], glucosamine [118], and CS [119] are widely investigated ones. β-CD-based polyester was used to coat Gd-NPs and targeted by folic acid (FA) to develop novel targeted MRI CAs. The developed Gd-NPs@ β-CD–FA MRI CAs revealed no significant cytotoxicity and possessed high biocompatibility up to 500 µg concentration of Gd^3+^/mL. In vitro MRI experiments revealed targeted contrast T_1_ and T_2_ weighted MR imaging [120]. Bony et al. synthesized D-glucuronic acid coated Ln/Mn (Ln = Gd and Dy) oxide NPs (d_avg_ = 2.0 nm). The D-glucuronic acid coated Gd-NPs displayed strong positive contrast (T_1_ MRI) enhancements in 1.5 T [121]. Dextran coated Gd-NPs synthesized through a one-pot synthesis approach were found to be highly water-dispersible and non-toxic as determined in a cellular cytotoxicity test. relaxivities r_1_ = 12.2 and r_2_ = 29.3 s^−1^·mM^−1^ with r_2_/r_1_ = 2.4 were observed [122]. In another study, dextran-coated Gd-phosphate NPs was employed both as tumor-targeted MRI CAs and as a vehicle for drug delivery to tumors [123]. CS has been widely used to functionalize the Gd-CAs owing to its biocompatibility, bioactivity, biodegradability, and non-toxicity. Recent studies reported hydrophilic and biocompatible chitosan oligosaccharide lactate (COL)-coated ultra-small Gd-NPs synthesized through a one-pot polyol method. The in vitro cellular cytotoxicity assay indicated that the COL-coated Gd-NPs were non-toxic up to 500 μM Gd and their r_1_ and r_2_ values were estimated to be 13.0 and 27.0 s^−1^·mM^−1^, respectively, which are higher than those of commercial MRI CAs [124]. 

### 4.3. Lipids and Their Derivatives

**Figure 3:**
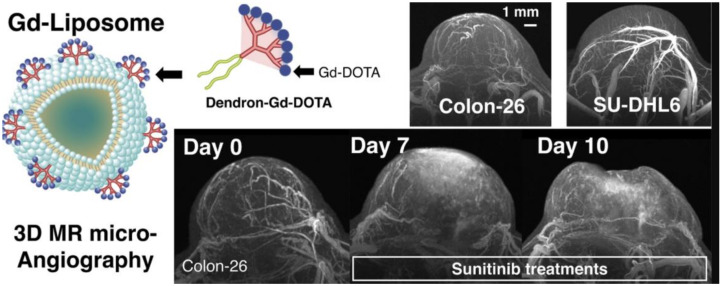
Gd-dendron assembled liposomal CAs, Images of Colon-26 and SU-DHL6 and Profiling of Colon-26 tumor vascular alterations via MR mA after anti-angiogenic therapy, 7 and 10 days after daily treatment with sunitinib are shown. Adapted with permission from [127]. Copyright 2018 Elsevier.

Lipids and their derivatives, such as liposomes and choline, are used to functionalize Gd-CAs. Phospholipid liposomes impart biocompatibility and increase the functionality of the Gd-CAs [125]. Generally, Gd-chelates are encapsulated inside the liposomes to synthesize tumour-targeting Gd-CAs. Thermosensitive Gd-CAs were synthesized conjugating thermosensitive liposomes with several clinically approved Gd-CAs where the r_2_ value was observed to be increased in the temperature range from 38–44 °C [126]. In an effort to develop a high-resolution, 3D tumor evaluation process, a Gd-dendron assembled liposomal NPs CAs was synthesized. A clear image of the tumor micro-vessel structure using the 50-μm isotropic MR angiography was obtained using Gd-liposome. It also facilitated the observation of the differences in the vascular structures of malignant lymphoma grafted models (Colon-26 and SU-DHL6) and their therapeutic alterations using a chemotherapeutic drug (sunitinib) as shown in Figure 3 [127].

Choline is an essential nutrient that can easily break through the blood-brain barrier (BBB) and is used to design targeted imaging agents for diagnosing brain tumors by MRI [128]. Lattuada et al. synthesized the Gd-DTPA-cholesterol complex, which can easily incorporate inside the mixed micelles yielding MRI CAs with increased relaxivity having applications in magnetic resonance angiography (MRA) [129]. Lipoproteins are spherical macromolecular particles consisting of a hydrophobic core and is surrounded by apolipoproteins and cholesterols. Highly biocompatible recombinant lipoprotein-like NPs were used in several studies acting as drug delivery vehicles and diagnostic agents. Rui et al. synthesized two different liver-specific MRI CAs conjugating Gd with cholesterol yielding Gd-DTPA-labeled cholesterol-containing recombinant high-density lipoprotein (HDL)-NPs, i.e., Gd-cholesterol-HDL and Gd-(cholesterol)_2_-HDL. The synthesized HDL-NPs provided signal enhancement in the liver [130]. 

### 4.4. Synthetic Polymers

**Figure 4:**
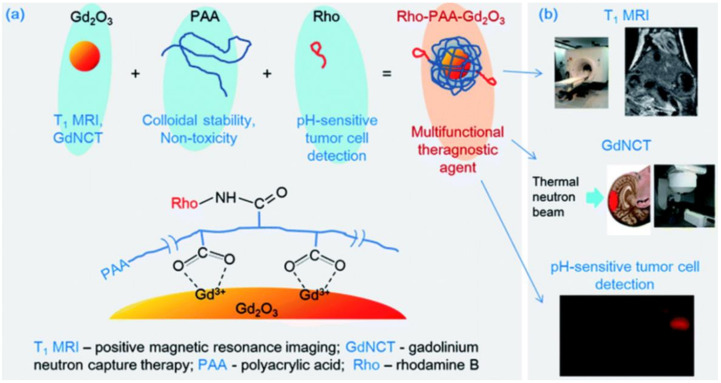
(**a**) Three components (i.e., Gd-NPs, PAA, and Rho) of the ultrasmall Gd-NPs colloid, the role of each component, and the surface coating structure. (**b**) Three applications of the ultrasmall Gd-NPs colloid. Reprinted from [135].

Several synthetic polymers (degradable and non-degradable) have been extensively used to synthesize modified Gd-CAs. Generally, these are synthesized by conjugation, encapsulation and chelation. Degradable synthetic polymers are often preferred owing to their biocompatibility and intrinsic biodegradability, while non-degradable synthetic polymers possessing hydrophilicity are an abundant choice. Poly(amino acid)s (PAAs) are commonly employed to synthesize conjugated Gd-CAs having improved relaxivity, biocompatibility, and biodegradability [131]. Miao et al. reported the synthesis of stable and non-toxic Gd-NPs colloids coated with PAAs as positive T_1_ MRI-CAs having optimum particle diameter size for renal excretion. Additionally, they possessed a high r_1_ value of 31.0 ± 0.1 s^−1^·mM^−1^ and r_2_/r_1_ ratio of 1.2, where r_1_ was approximately eight times higher than that of commercial Gd-chelates. High positive contrast enhancements were observed in the liver, kidneys and bladder of the mouse [113]. Poly(ethylene glycol) (PEG) containing PAAs block copolymers (b-poly) are used to functionalize Gd-CAs. In a study, Gd-DOTA-based CAs were synthesized by conjugating Gd(III) ions to PEG-b-poly(L-lysine)/(PEG-b-P(Lys)) through the ligand DOTA. Synthesized complex PEG-P(Lys-DOTA-Gd displayed r_1_ = 5.6–7.3 s^−1^·mM^−1^ at 9.4 T [132]. A novel theragnostic vesicle exhibiting admirable T_1_-weighted MRI contrast effect was developed by conjugating BSA-Gd with amphiphilic di-block copolymer poly(ethylene glycol)-block-poly(L-lactic-co-glycolic acid) (PEG-b-PLGA) [133]. In a similar approach, novel multifunctional polymeric CAs were synthesized by conjugating polylactic acid/PEG (PLA/PEG)-P(Lys)/poly(lactic acid) (PLA) with anti-vascular endothelial growth factor (VEGF) antibody and Gd-DTPA yielding Anti-VEGF-PLA-PEG-PLL-Gd-NPs) for the targeted delivery of Gd-DTPA to the liver cancer. The Anti-VEGF PLA-PEG-PLL-Gd-NPs displayed high T_1_ relaxivity and no apparent cytotoxicity in human liver cancer (HepG2) cells under experimental concentrations [134]. Ho et al. developed monodispersed Gd-NPs colloids (d_avg_ = 1.5 nm) coated with hydrophilic polyacrylic acid (PAA) and partly conjugated with rhodamine B (Rho) for an additional functionalization (mole ratio of PAA: Rho = 5:1). The NPs colloids exhibited a very high r_1_ of 22.6 s^−1^·mM^−1^ (r_2_/r_1_ = 1.3), which was ~6 times higher than those of commercial Gd-chelates. These NPs colloids were applied to Gd-NCT in vitro and exhibited a significant U87MG tumor cell death (28.1% net value) after thermal neutron beam irradiation, which was 1.75 times higher than that obtained using commercial Gadovist. These colloids also exhibited stronger fluorescent intensities in tumor cells than in normal cells owing to conjugated Rho, proving their pH-sensitive fluorescent tumor cell detection ability (Figure 4) [135].

Jang and coworkers synthesized poly(acrylic acid-co-maleic acid) (PAAMA) coated Gd-NPs (d_avg_ = 1.8 nm), which exhibited outstanding colloidal stability, exceptionally low cellular toxicity, and a high r_1_ value of 40.6 s^−1^·mM^−1^ and r_2_/r_1_ ratio of 1.56, which is approximately 10 fold higher than those of commercial molecular CAs [136]. In another study, poly(methyl vinyl ether-alt-maleic acid) (PMVEMA) was used as a surface-coating polymer to develop PMVEMA-coated Gd-NPs, which displayed excellent colloidal stability in aqueous solution and appreciable biocompatibility. The r_1_ value of 36.2 s^−1^·mM^−1^ and r_2_/r_1_ = 2 under a 3 T MR field was observed (approximately 10 fold higher value than that of commercial molecular CAs) [137]. 

**Figure 5:**
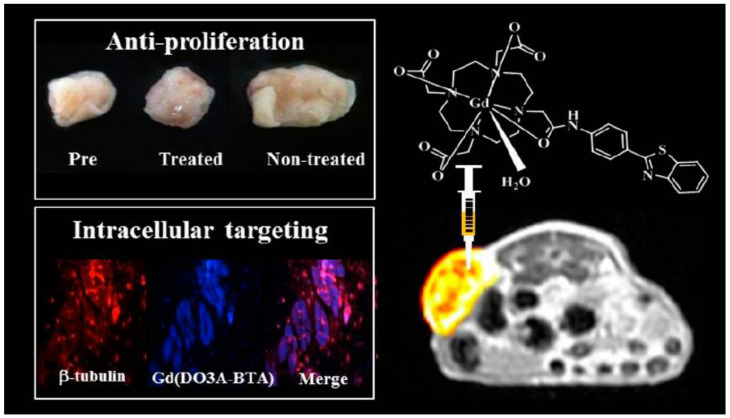
Antiproliferative activity and intracellular targeting of [Gd(DO3A-BTAA)(H_2_O)] complex. Reprinted with permission from [142]. Copyright 2013 American Chemical Society.

In a recent study, hybrid polyion complexes (HPICs) were synthesized by mixing the metal ions, zirconium ion (ZrO^2+^) combined with Gd^3+^ ions to a double-hydrophilic poly(ethylene oxide) (PEO)-b-PAA block copolymer solution forming nanostructures with an average radius of 11–16 nm. An increase in the zirconyl mole fraction imparted excellent chemical stability, while the addition of Gd^3+^ greatly enhanced magnetic relaxivity. This behavior presents these nanostructures as excellent CAs having good biocompatibility [138]. In order to achieve a multimodal theragnostic agent, Hu et al. developed a water-soluble Gd containing polymer-based theragnostic compound (PFTQ-PEG-Gd-NPs) for the in vivo tri-modal photoacoustic imaging (PA)/MR second near-infrared (NIR-II) imaging-guided tumor photothermal therapy (PTT). PFTQ is a semiconducting conjugated polymer synthesized through a grafting-on method by utilizing thiadiazoloquinoxaline (TQ) and fluorene (F) as the acceptor and donor, respectively, to endow the complex with near-infrared (NIR) absorption and fluorescence emission capabilities. These agents possessed low biotoxicity and outstanding chemical and optical stability. Administration of PQTF-PEG-Gd-NPs in the tumorous sites of the in vivo model of mice displayed enhancement in PA, NIR-II fluorescence and positive MR signal intensities after 24 h of systemic administration [139]. Grogna and coworkers synthesized Gd^3+^ based MRI CAs by grafting 1,4,7,10-tetraazacyclododecane-1,4,7-triacetic acid, 1,4,7-tris(1,1-dimethyl ethyl) ester (DO3AtBu) onto succinimidyl esters and PEO chains. DO3AtBu-NH_2_ was synthesized by dissolving DOTA(tert-butyl ester)-methylic ester in ethylenediamine. This was grafted onto the copolymers bearing activated esters, i.e., succinimidyl esters and PEO chains. Gd^3+^ ions were then attached to unprotected carboxyl groups attached DO3AtBu-NH_2_ by stirring the GdCl_3_.6H_2_O solution with DO3AtBu-NH_2_ to yield the DO3A(Gd^3+^)-NH_2_ complex. The synthesized MRI CAs displayed enhanced relaxivity and efficient long blood circulation lifetime [140]. Kang et al. developed two types of Gd-NPs, i.e., Gd-NPs@SiO_2_-DO3A and Gd-NPs@SiO_2_-DO2A-benzothiazoles (BTA) with diameters of 50–60 nm. The synthesis involved sequential coating of Gd-NPs with tetraethyl orthosilicate (TEOS) and 3-aminopropyl triethoxysilane (APTES), followed by functionalization of the aminopropyl silane group with DOTA or 1,4,7,10-tetraazacyclododecane-1,4,7-trisacetic acid (DO3A) conjugates of BTA (DO3A-BTA). Gd-NPs@SiO_2_-DO3A and Gd-NPs@SiO_2_-DO2A-BTA exhibited high water solubility and colloidal stability. The r_1_ relaxivities of both Gd-NPs@SiO_2_-DO3A and Gd-NPs@SiO_2_-DO2A-BTA were found to be higher than those of the corresponding low-molecular-weight MRI CAs, and their r_2_/r_1_ ratios were close to 1, indicating that both can be used as potential T_1_ MRI CAs. Biodistribution studies demonstrated that Gd-NPs@SiO_2_-DO2A-BTA was excreted via both hepatobiliary and renal pathways. Gd-NPs@SiO_2_-DO2A-BTA exhibited a strong intracellular uptake property in a series of tumor cell lines and had significant anticancer characteristics against cell lines such as SK-HEP-1, MDA-MB-231, HeLa, and Hep-3B [141]. A Gd complex of DO3A and BTA-aniline (BTAA) of the type [Gd(DO_3_A-BTAA)(H_2_O)] was reported by Kim et al. for use as a single molecule theragnostic agent. The kinetic inertness and r_1_ relaxivity (3.84 s^−1^·mM^−1^) of the complex compared well with those of structurally similar analogous Gd-DOTA. The same complex was found to be tumor-specific, and intracellular enhanced MR images of cytosols and nuclei of tumor cells such as MCF-7, MDA-MB-231, and SK-HEP-1 were observed. Both DO3A-BTAA and Gd(DO3A-BTAA) revealed antiproliferative activities (Figure 5) [142].

### 4.5. Organic and Inorganic Molecules

In spite of the employment of several polymers and lipids for the conjugation of Gd-CAs, several organic and inorganic molecules such as chalcones (Chal), fluorescein, carbon, fullerene, GO, silica, and Au have also found their application in imparting enhancement in bioimaging applications. Gd-CAs have been conjugated with several organic and inorganic materials such as GO, silica, Chal, and other NPs to provide additional efficacy. 

Carbon is an ideal surface-coating material on NPs for promoting biomedical applications owing to its biocompatibility, nearly chemically inertness, and photoluminescent properties in the visible region. Carbon-coated Gd-NPs were synthesized by Yue and coworkers in an aqueous solution using a simple method. Gd-NPs@C (d_avg_ = 3.1 nm) displayed excellent colloidal stability, very high r_1_ value (16.26 s^−1^·mM^−1^; r_2_/r_1_ = 1.48) and exhibited photoluminescence in the visible region. In vivo positive (T_1_) MR images of high contrast indicated that the Gd-NPs@C could prove to be a potential T_1_ MRI CAs. Additionally, strong fluorescence in the visible region was observed due to carbon coating on the NPs surfaces, indicating that the synthesized materials are eligible for a dual-modal imaging agent [143]. The GO and fullerenes have been employed to functionalize Gd-CAs for MRI and drug delivery applications. A water dispersible Gd-NPs decorated with GO nanocomposites (NCs) (Gd-NPs/GO-NCs) was developed through a simple solvent evaporation process. The Gd-NPs/GO-NCs displayed a high relaxivity value of 34.48 s^−1^·mM^−1^ with good biocompatibility [144]. The GQDs, derivatives of graphene exhibit promising applications in the field of biosensors, drug/gene delivery vehicles, and bioimaging agents owing to their easy functionalization, biocompatibility, and intrinsic fluorescence properties. Li et al. developed Mn^2+^ or Gd^3+^ doped nitrogen-containing GQDs (NGQDs) as biocompatible MRI CAs. Synthesis was performed through a single-step microwave-assisted hydrothermal reaction to get Mn-NGQDs and Gd-NGQDs having dual MRI and fluorescence modalities. The synthesized quasi-spherical 3.9–6.6 nm average-sized nanostructures possessed highly crystalline graphitic lattice structures (0.24 and 0.53 atomic% for Mn^2+^ and Gd^3+^ doping). These structures possessed high in vitro biocompatibility with values up to 1.3 mg·mL^−1^ for Mn-NGQDs and 1.5 mg·mL^−1^ for Gd-NGQDs, having effective internalization in HEK-293 cells traced by intrinsic NGQD fluorescence. Mn-NGQDs exhibited substantial r_2_/r_1_ ratios of 11.190. At the same time, Gd-NGQDs possess r_2_/r_1_ of 1.148 with a high r_1_ value of 9.546 s^−1^·mM^−1^ These results demonstrate their potential for not only biocompatible alternatives to available T_1_/T_2_ and T_1_ CAs but also as FI agents [145]. Gd^3+^ ions loaded PEG-modified Gd-PEG-GO-QDs were synthesized where the r_1_ was effectively enhanced by increasing the proton exchange. A 20–30 folds increase in r_1_ values of Gd-PEG-GO-QDs was observed as compared to the commercial CAs. Additionally, Gd-PEG-GO-QDs possessed low biotoxicities while the FA modified Gd-PEG-GO-QDs demonstrated efficiency for MRI-fluorescence bimodal tumor targeting agent in animals having greater than 98.3% specific cellular uptake rate [146].

The chemical inertness, biocompatibility, non-toxicity, stability, and magnetism transparency of silica has made it a favourable choice for the conjugating Gd-CAs. A new strategy to enhance the relaxivity and image contrast was developed by inserting Gd-CAs inside the nanoporous silicon NPs [68]. Theragnostic agents having therapeutic and imaging modalities are prepared by the development of theragnostic nano-vectors having the ability to release therapeutic agents at the pathological sites and provide instantaneous MR images. Carniato et al. developed Gd based MSNPs by selectively functionalizing the external surface of MSNPs loaded with ibuprofen molecules and functionalized with Gd–DOTA-monoamide chelate for the relaxivity and drug release studies of the Gd based MSNPs [147]. In another effort, DOX (an anticancer drug) was incorporated with MSNPs along with Gd_2_O_3_ forming Gd-NPs@MSN-DOX. These hybrid NPs were coated with pH-responsive polyelectrolytes, which, upon entering into cells, disassociated from the surfaces of the Gd-NPs @MSN-DOX and thus activated the DOX release [148]. A high relaxivity Au-based bimodal Gd-CAs were synthesized and investigated for the effects of shape on proton relaxation. Gd^3+^were covalently attached to thiolated DNA, forming the nanoconjugates (Gd-DNA), followed by the conjugation onto Au nanostars (DNA-Gd@stars), which displayed efficient Gd(III) delivery and biocompatibility [76]. Liu and others synthesized Gd(III))- dendrimer-AuNPs (AuNPs-DEN) as multimodal CT/MRI agents. Poly(amidoamine) (PAMAM) dendrimers were partly conjugated with carboxybetanie acrylamide (CBAA), 2-methacryloyloxyethyl phosphorylcholine (MPC), and 1,3-propane sultone (1,3-PS), respectively, and then the Au NPs were entrapped within while the remaining amine terminal was covered by acetylation. Zwitterionic Gd(III)-loaded AuNPs-DEN modified with arginine-glycine-aspartic acid peptide were then synthesized for targeted dual-mode CT/MR imaging. It was observed that AuNP-DEN (AuNPs core size of 2.7 nm and a surface potential of 7.6 ± 0.9 mV) displayed good X-ray attenuation properties, relatively higher r_1_ values (13.17 s^−1^·mM^−1^), satisfactory cytocompatibility, and targeting specificity to α_v_β_3_ integrin-expressing cancer cells [149]. In another study, mixed Gd-dysprosium oxide nanoparticles (Gd-DONPs) were synthesized as a dual-mode T_1_ and T_2_ MRI contrast agent. The D-glucuronic acid coated Gd-DONPs (d_avg_ = 1.0 nm) exhibited large r_1_ and r_2_ values (r_2_/r_1_ ≈ 6.6), displaying obvious dose-dependent contrast enhancements in R_1_ and R_2_ map images. In vivo T_1_ and T_2_ MR images revealed the dual-mode imaging capability of the NPs [150]. Choi et al. developed surface-doped with manganese oxide (MnO) Gd-NPs abbreviated as Gd-NPs@MnO ranging from 1 to 2 nm in diameter, coated with hydrophilic biocompatible compound, lactobionic acid (LA). In-vitro studies revealed dose-dependent contrast enhancements in both T_1_ and T_2_ map images, establishing their potential as both T_1_ and T_2_ MRI CAs [151]. Mixed Zn(II)/Gd(III) oxide NPs with approximately 8 mole% of Zn with an average diameter of 2.1 nm were synthesized by Tegafaw et al. in their studies. The D-glucuronic acid coated Zn(II)/Gd(III) oxide NPs showed a r_1_ value of 12.3 s^−1^·mM^−1^ with r_2_/r_1_ ratio of 1.1, corresponding to an ideal condition for T_1_ MRI CAs. This could be attributed to the reduced magnetization of the mixed NPs because of non-magnetic Zn in the NPs [152]. A LA coated Gd–europium oxide NPs (d_avg_ 1.75 nm) with good water solubility and biocompatibility were synthesized and evaluated for T_1_, T_2_ MRI-FI in vitro and in vivo. The r_1_ value of 11.9 and r_2_ values of 38.7 s^−1^·mM^−1^ were observed, displaying clear dose-dependent contrast images. In addition, they showed both positive and negative contrast enhancements in 3 T and fluorescent confocal images in both DU145 cells and *C*. *elegans* (a small nematode). This study demonstrates the T_1_, T_2_ MRI-FI multi-functionality of LA coated mixed Gd–europium oxide nanoparticles [153].

Kim et al. reported a new concept of neuroprognostic agents, which combines molecular diagnostic imaging and targeted neuroprotection to treat reperfusion injury after stroke. These neuroprognostic agents are inflammation-targeted Gd compounds conjugated with nonsteroidal anti-inflammatory drugs (NSAIDs). It was found that Gd-based MRI CAs conjugated with NSAIDs suppressed the increase in cyclooxygenase-2 (COX-2) levels, ameliorated glial activation, and neuron damage that is phenotypic for stroke by mitigating neuroinflammation, which prevented reperfusion injury. In addition, it was also observed that the neuroprognostic agents were promising T_1_ molecular MRI CAs for detecting precise reperfusion injury locations at the molecular level [154]. Multifunctional imaging of the deposition of amyloid-beta (Aβ) aggregates in the brain is of great importance in diagnosing Alzheimer’s disease. A novel multifunctional Aβ-targeting Gd-CAs comprised of Gd-chelate conjugated with Chal (Gd-DO3A-Chal) was synthesized by Choi and coworkers. Studies revealed that Gd-DO3A-Chal showed 8 times higher binding affinity to Aβ aggregates than a previously reported Gd-chelate conjugated with Pittsburgh compound B. Gd-DO3A-Chal showed multimodal imaging capability [155]. Park et al. utilized organic molecules for the surface coating Gd-NPs where all the samples exhibited large r_1_ and r_2_ water proton relaxivities with r_2_/r_1_ ratios that were close to one, corresponding to ideal conditions for T_1_ MRI CAs. Finally, in-vivo T_1_ MR images were acquired to prove the effectiveness of the surface-coated ultrasmall Gd-NPs as T_1_ MRI CAs [156]. Baek et al. synthesized a macrocyclic Gd chelate based on DO3A coordination cage having an ethoxybenzyl (EOB) moiety and studied it as a T_1_ hepatobiliary MRI contrast agent. Synthesized agents displayed high chelation stability and high r_1_ relaxivity as compared with the linear-type Gd chelates (currently clinically approved liver agents). In addition, they also displayed high tumor detection sensitivity [157]. Fluorescein is an organic compound and dye and is most frequently used in optical contrast media for the synthesis of organic dyes-based MRI/OI dual-modal Gd-CAs. Miao et al. synthesized a dye-coated Gd-NPs [dye = fluorescein and fluorescein isothiocyanate (FITC)] in a one-pot synthesis approach and investigated their dual imaging properties. The dye-coated Gd-NPs exhibited excellent relaxometric properties suitable for T_1_ MRI: r_1_ = 9.8 s^−1^·mM^−1^ (r_2_/r_1_ = 2.6) for fluorescein-coated NPs (d_avg_ = 1.6 ± 0.1 nm) and r_1_ = 12.3 s^−1^·mM^−1^ (r_2_/r_1_ = 2.3) for FITC-coated NPs (d_avg_ = 1.4 ± 0.1 nm), and strong photoluminescence (PL) in the green region (around 514 nm) suitable for FI. The dye-coated NPs exhibited strong fluorescence in cellular confocal images and high contrast in T_1_ MR images in mice, suggesting that they are potential dual T_1_ MRI-FI agents [158,159]. Macrocyclic diethylenetriamine penta-acetic acid (DTPA) conjugates of 2,2′-diaminobiphenyl and their Gd complexes were synthesized and studied for their potential use as new MRI blood-pool CAs (MRI-BPCAs). The r_1_ relaxivity values were found to be 10.9 s^−1^·mM^−1^, which is approximately 3 times as high as that of structurally related Gd-DOTA (r_1_ = 3.7 s^−1^·mM^−^^1^). The r_1_ relaxivity in human serum albumin (HSA) goes up to 37.2 s^−1^·mM^−1^, almost twice as high as that of MS-325, a leading BPCAs, demonstrating a strong blood pool effect [160]. In a similar approach, Gd-complexes consisting of DOTA conjugates of tranexamic acid and tranexamic esters were prepared as a new class of MRI-BPCAs. The r_1_-relaxivity was significantly higher than those of any of the clinically used MRI CAs [161]. Table 3 briefly summarizes r_1_, particle size, biotoxicity, specific properties and modality of Gd-CAs.

## 5. Conclusions and Future Perspectives

Gd-CAs have been widely studied for their magnetic properties and have been utilized as positive MRI CAs. In this review, we have outlined the basic imaging techniques and their limitations. Functionalization of Gd-CAs from single-modality to multi-modality imaging has emerged as a major aspect in overcoming the limitations of current commercially available CAs. Currently, major publications are based on the application of the NPs on the enhancement of relaxivity values and biocompatibility of the designed Gd-CAs. Although several studies have reported, agents having relaxivities higher than that of commercially available CAs, the prospect of the potential toxicity of nanomaterials and probable risk of release of Gd limits their clinical applications. Factors such as the size and shape of NPs, and their surface charge and surface labelling impact the biodistribution of NPs. Encapsulation and surface labelling can reduce the toxic effects of synthesized NPs, but still, more studies are required to understand these aspects. Therefore, the development of Gd-CAs conjugated with ultra-small NPs having rapid elimination can somewhat overcome their toxicity issue. Imparting multi-modality to Gd-CAs has become a fascinating ideology, and it has been widely accepted as the next generation CAs. Due to the outstanding MRI enhancement characteristic of Gd, Gd-CAs can be coupled with other modalities to enhance or complement their imaging abilities. CT imaging, most widely used to afford detailed information of skeletons, lacks in yielding information on soft tissues, whereas MRI can offer high spatial anatomic information of tissues. Therefore Gd-CAs having the combination of MRI and CT could be of great significance in clinical applications. Gd-CAs augmented with optical imaging CAs complements their applications. PET can be incorporated with MR imaging of Gd-CAs to yield high-resolution images with anatomical details. The Gd agents for theragnostics have a potential role that incorporates diagnostics with therapeutics in the same platform. This strategy involves targeted delivery, controlled release and simultaneous diagnosis and treatment at the molecular level. Smart carriers or delivery vehicles NPs encapsulate various therapeutic agents and deliver them to target locations at the time of bioimaging. 

In spite of these developments, most of the CAs failed to attain clinical applications, and only a handful of CAs have reached clinical trials. Current studies should be more focused on target specificity and reduced toxicity, exploring the Gd NPs to their full potential. Gd-CAs can be conjugated with highly specific tumor-targeting ligands, such as antibodies, aptamers, and peptides. Second, to increase the biocompatibility, renal excretion and non-toxicity, Gd-CAs should be conjugated to hydrophilic and biocompatible ligands, small enough to be excreted through the renal system. In addition, understanding the pharmacokinetics of these agents in humans should be emphasized. Effective development of Gd-CAs for bioimaging applications will require multidisciplinary research efforts. Despite these challenges, developing such Gd-CAs can prove to be a breakthrough in bioimaging CAs in the future.

## Figures and Tables

**Table 1:** A summary of bioimaging modalities in clinical use [7,8].

Imaging Technique	Detection	Imaging Probes	Common CAs	Some Clinical Applications	Advantages	Disadvantages
Optical imaging	Visible, ultraviolet, and infrared light	Organic dyes, QDs, Lanthanide ion	Fluorescein, cresyl violet acetate, indocyanine green, toluidine blue	Optical microscopy, endoscopy, scanning laser ophthalmoscopy	High sensitivity	Low resolution and poor tissue penetration
Computed tomography	X-rays	Iodine, Lanthanide, Gold compounds	Iopamidol, ioxaglate	Cerebral infarction, angiography	High spatial resolution	Costly with poor soft tissue imaging
Magnetic resonance imaging	Magnetic field	Gd, Fe, Mn compounds	Gadoteridol, gadopentetate dimeglumine	Cerebral and coronary angiography	High-resolution Excellent signal in soft tissues	Costly with low sensitivity
Positron emission tomography	Gamma-rays	Radioactive elements: ^18^F, ^15^O, ^64^Cu ^68^Ga etc.	^18^FDG, ^15^H_2_O, ^68^Ga-EDTA, ^11^C-methionine	Degenerative diseases and cerebral blood flow	Quantitative	Exposure to radiation with poor resolution
Single photon emission computed tomography	Gamma-rays	Radioactive elements: ^18^F, ^11^C, ^15^O, ^68^Ga, ^64^Cu etc.	^99^mTc-HMPAO, ^99^mTc-ECD, ^111^In-octreotide	Dementia, cardiac imaging and cerebral infarction	Quantitative	Exposure to radiation with poor resolution
Ultra- sonography	Ultrasonic waves	Microbubbles	Microbubbles	Congenital conditions and echocardiography	Cost-effective, simple, and fast.	Poor resolution

^18^FDG: Radiotracer, F-18 fluorodeoxyglucose. ^68^Ga-EDTA: 68Ga-labeled ethylenediaminetetraacetic acid. ^99^mTc-HMPAO: technetium-99m-D,L-hexamethylpropylene amine oxime. ^99^mTc-ECD: 99mTc-ethylcysteinate dimer.

**Table 2:** Gd-CAs for imaging and therapy.

Imaging Modality	Nanoparticles *	Applications	Ref.
MRI	Liposomal Gd	Imaging placenta	[75]
MRI	Gd(III)-thiolated DNA–Au nanostars (DNA-Gd@stars)	Imaging pancreatic cancer cells	[76]
MRI/CT	Gd(III)-decorated Au NPs	Enhancing the relaxometric properties of Gd(iii) complexes	[77]
MRI/OI	GQDs-folate-DOX-Gd	Bioimaging and tumor targeted drug delivery	[78]
MRI/PET	Pegylated liposome (LP)-(Gd)-positron-emitting 89Zr	Imaging of Cancer	[79]
MRI/SPECT	Gd Complex of 125I/127I-RGD-DOTA Conjugate	Tumor targeting	[80]
MRI/US	Liposomes-Gd-rhodamine	MRI Monitoring and Quantification of US-Mediated Delivery	[81]
NIRF/CT/MRI	Gold–Gd nanoclusters (NCs)	Tumor targeting and low body residues	[82]
MRI/US/CT	Gd- Gold Microcapsules	Multimodal cellular imaging of transplanted islet cells	[83]
MRI/CT/PAI	Bismuth-Gd-PEG NPs	Imaging-guided photothermal cancer therapy	[84]

* DNA: Deoxyribonucleic acid. Au: Gold. DOTA: 1,4,7,10-tetraazacyclododecane-1,4,7,10-tetraacetic acid. RGD: Arginine-Glycine-Aspartic acid tripeptide. GQDs: Graphene quantum dots. DOX: Doxorubicin. PAI: Photoacoustic Imaging.

**Table 3:** Summary of magnetic relaxivity (r_1_), particle size (d_avg_), specific properties and modalities of reported CAs.

S. No.	CAs	Magnetic Relaxivity (r_1_) (s^−1^·mM^−1^)	Particle Size (d_avg_) (nm)	Specific Characteristics	Modality	Ref.
1	Gd–albumin conjugates	9~10.5	~5–6	Blood clearance half-lives = 40–47 min	T_1_ MRI	[110]
2	Gd–albumin-Folic acid conjugates	10.8	~201–215	Almost non-cytotoxic, good biocompatibility	T_1_ MRI	[111]
3	TAT-Gd-NPs	18.2	1.5	Good in vitro cell viability, non-toxic upto 20 μM Gd	T_1_ MRI	[112]
4	Cyclic RGD-conjugated Gd-NPs	10.0–18.7	1.0–2.5	Nontoxic up to 10 μM Gd	T_1_ MRI	[25]
5	PPy@BSA-Gd	10.203	50	Good cytocompatibility, Phototherma therapy	T_1_ MRI	[114]
6	D-glucuronic acid coated Gd-NPs	12.2	2.0	Highly water-dispersible and non-toxic	T_1_ MRI	[122]
7	Chitosan oligosaccharide lactate (COL)-Gd-NPs	13.0	1.9	Non-toxic up to 500 μM Gd	T_1_ MRI	[124]
8	Gd-NPs-polyacrylic acid (PAA)-rhodamine B (Rho)	22.6	1.5	High cell viabilities up to 500 μM Gd and good biocompatibility	T_1_ MRI-NCT-FI	[135]
9	PAAMA coated Gd-NPs	40.6	1.8	Exceptionally low cellular toxicity	T_1_ MRI	[136]
10	PMVEMA-coated Gd-NPs	36.2	1.9	Excellent colloidal stability in aqueous solution and appreciable biocompatibility	T_1_ MRI	[137]
11	PFTQ-PEG-Gd-NPs	10.95	95 ± 4.6	Low biotoxicity and outstanding chemical and optical stability	T_1_ MRI	[139]
12	Gd-NPs@SiO_2_-DO3A and Gd-NPs@SiO_2_-DO2A-benzothiazoles (BTA)	Gd@SiO_2_-DO3A = 5.47 Gd@SiO_2_-DO2A-BTA = 7.99	50–60	High water solubility and colloidal stability, anticancer characteristics	T_1_ MRI	[141]
13	Gd(DO3A-BTAA)(H_2_O) chelates	3.84	-	Tumour-specific, antiproliferative activities	T_1_ MRI	[142]
14	Gd-NPs@C	16.26	3.1	Cell viabilities up to 500 μM Gd, good biocompatibility	T_1_ MRI-FI	[143]
15	Gd-NPs/GO-NCs	34.48	2.89	Water dispersible with good biocompatibility	T_1_ MRI	[144]
16	Gd-PEG-GO-QDs	210.9 at 114 μT	4.0	Low biotoxicities	T_1_ MRI-FI	[146]
17	Gd(III)-loaded AuNPs-DEN modified with arginine-glycine-aspartic acid peptide Complex	13.17	-	Satisfactory cytocompatibility	T_1_ MRI-CT	[149]
18	Gd-dysprosium oxide nanoparticles (Gd-DONPs) D-glucuronic acid coated Gd-DONPs	6.0	1.0	Non-toxic up to 200 μM	T_1_, T_2_ MRI	[150]
19	D-glucuronic acid coated Zn(II)/Gd(III) oxide NPs	12.3	2.1	Slightly cytotoxic in DU145 cell lines, negligible cytotoxicity in NCTC1469 cell lines up to 200 M (Gd + Zn)	T_1_ MRI	[152]
20	LA coated Gd–europium oxide NPs	11.9	1.75	Non-toxic up to 279 mM Gd and Eu	T_1_,T_2_ MRI-FI	[153]
21	Gd compounds –NSAIDs chelates	5.0–7.0	-	Neuroprognostic	T_1_ MRI	[154]
22	Gd-chelate conjugated with Chal (Gd-DO3A-Chal	4.95	-	Aβ-targeting	MRI-FI	[155]
23	Fluorescein/FITC-Gd-NPs	Fluorescein-coated = 9.8 FITC-coated = 12.3	Fluorescein-coated = 1.6 FITC-coated = 1.4	Good cell viability up to 100 μM Gd	T_1_ MRI-FI	[158]

## Data Availability

Not applicable.

## References

[1] Ferrari M. (2005). Cancer nanotechnology: Opportunities and challenges. Nat. Rev. Cancer.

[2] Brown M.A., Semelka R.C. (2011). MRI: Basic Principles and Applications.

[3] Weissleder R. (2006). Molecular imaging in cancer. Science.

[4] Greish K. (2007). Enhanced permeability and retention of macromolecular drugs in solid tumors: A royal gate for targeted anticancer nanomedicines. J. Drug Target..

[5] Hawley A.E., Illum L., Davis S.S. (1997). Preparation of biodegradable, surface engineered PLGA nanospheres with enhanced lymphatic drainage and lymph node uptake. Pharm. Res..

[6] Davis M.E., Chen Z., Shin D.M. (2010). Nanoparticle therapeutics: An emerging treatment modality for cancer. Nanosci. Technol. A Collect. Rev. Nat. J..

[7] Sharma P., Brown S., Walter G., Santra S., Moudgil B. (2006). Nanoparticles for bioimaging. Adv. Colloid Interface Sci..

[8] Zhang L., Liu R., Peng H., Li P., Xu Z., Whittaker A.K. (2016). The evolution of gadolinium based contrast agents: From single-modality to multi-modality. Nanoscale.

[9] Donato H., França M., Candelária I., Caseiro-Alves F. (2017). Liver MRI: From basic protocol to advanced techniques. Eur. J. Radiol..

[10] Foucault-Collet A., Gogick K.A., White K.A., Villette S., Pallier A., Collet G., Kieda C., Li T., Geib S.J., Rosi N.L. (2013). Lanthanide near infrared imaging in living cells with Yb3+ nano metal organic frameworks. Proc. Natl. Acad. Sci. USA.

[11] Cai Z., Ye Z., Yang X., Chang Y., Wang H., Liu Y., Cao A. (2011). Encapsulated enhanced green fluorescence protein in silica nanoparticle for cellular imaging. Nanoscale.

[12] Genovese D., Bonacchi S., Juris R., Montalti M., Prodi L., Rampazzo E., Zaccheroni N. (2013). Prevention of self-quenching in fluorescent silica nanoparticles by efficient energy transfer. Angew. Chem. Int. Ed..

[13] Grebenik E.A., Nadort A., Generalova A.N., Nechaev A.V., Sreenivasan V.K.A., Khaydukov E.V., Semchishen V.A., Popov A.P., Sokolov V.I., Akhmanov A.S. (2013). Feasibility study of the optical imaging of a breast cancer lesion labeled with upconversion nanoparticle biocomplexes. J. Biomed. Opt..

[14] Lee S., Cha E., Park K., Lee S., Hong J., Sun I., Kim S.Y., Choi K., Kwon I.C., Kim K. (2008). A near-infrared-fluorescence-quenched gold-nanoparticle imaging probe for in vivo drug screening and protease activity determination. Angew. Chem..

[15] Muthukumar T., Chamundeeswari M., Prabhavathi S., Gurunathan B., Chandhuru J., Sastry T.P. (2014). Carbon nanoparticle from a natural source fabricated for folate receptor targeting, imaging and drug delivery application in A549 lung cancer cells. Eur. J. Pharm. Biopharm..

[16] Wang Y., Zhou K., Huang G., Hensley C., Huang X., Ma X., Zhao T., Sumer B.D., DeBerardinis R.J., Gao J. (2014). A nanoparticle-based strategy for the imaging of a broad range of tumours by nonlinear amplification of microenvironment signals. Nat. Mater..

[17] Markovic S., Belz J., Kumar R., Cormack R.A., Sridhar S., Niedre M. (2016). Near-infrared fluorescence imaging platform for quantifying in vivo nanoparticle diffusion from drug loaded implants. Int. J. Nanomed..

[18] Dubreil L., Leroux I., Ledevin M., Schleder C., Lagalice L., Lovo C., Fleurisson R., Passemard S., Kilin V., Gerber-Lemaire S. (2017). Multi-harmonic imaging in the second near-infrared window of nanoparticle-labeled stem cells as a monitoring tool in tissue depth. ACS Nano.

[19] Ghaghada K.B., Badea C.T., Karumbaiah L., Fettig N., Bellamkonda R.V., Johnson G.A., Annapragada A. (2011). Evaluation of tumor microenvironment in an animal model using a nanoparticle contrast agent in computed tomography imaging. Acad. Radiol..

[20] Bonitatibus P.J., Torres A.S., Goddard G.D., FitzGerald P.F., Kulkarni A.M. (2010). Synthesis, characterization, and computed tomography imaging of a tantalum oxide nanoparticle imaging agent. Chem. Commun..

[21] Hu Y., Wang Y., Jiang J., Han B., Zhang S., Li K., Ge S., Liu Y. (2016). Preparation and characterization of novel perfluorooctyl bromide nanoparticle as ultrasound contrast agent via layer-by-layer self-assembly for folate-receptor-mediated tumor imaging. Biomed. Res. Int..

[22] Wang X., Chen H., Zheng Y., Ma M., Chen Y., Zhang K., Zeng D., Shi J. (2013). Au-nanoparticle coated mesoporous silica nanocapsule-based multifunctional platform for ultrasound mediated imaging, cytoclasis and tumor ablation. Biomaterials.

[23] Seo M., Gorelikov I., Williams R., Matsuura N. (2010). Microfluidic assembly of monodisperse, nanoparticle-incorporated perfluorocarbon microbubbles for medical imaging and therapy. Langmuir.

[24] Pressly E.D., Pierce R.A., Connal L.A., Hawker C.J., Liu Y. (2013). Nanoparticle PET/CT imaging of natriuretic peptide clearance receptor in prostate cancer. Bioconjug. Chem..

[25] Ahmad M.Y., Ahmad M.W., Cha H., Oh I., Tegafaw T., Miao X., Ho S.L., Marasini S., Ghazanfari A., Yue H. (2018). Cyclic RGD-coated ultrasmall Gd_2_O_3_ nanoparticles as tumor-targeting positive magnetic resonance imaging contrast agents. Eur. J. Inorg. Chem..

[26] Petrik M., Weigel C., Kirsch M., Hosten N. (2005). No detectable nephrotoxic side effect using a dimer, non-ionic contrast media in cerebral perfusion computed tomography in case of suspected brain ischemia. RoFo Fortschr. Geb. Rontgenstrahlen Nukl..

[27] Oh I., Min H.S., Li L., Tran T.H., Lee Y., Kwon I.C., Choi K., Kim K., Huh K.M. (2013). Cancer cell-specific photoactivity of pheophorbide a–glycol chitosan nanoparticles for photodynamic therapy in tumor-bearing mice. Biomaterials.

[28] Hoshyar N., Gray S., Han H., Bao G. (2016). The effect of nanoparticle size on in vivo pharmacokinetics and cellular interaction. Nanomedicine.

[29] Scott R.P., Quaggin S.E. (2015). The cell biology of renal filtration. J. Cell Biol..

[30] Longmire M., Choyke P.L., Kobayashi H. (2008). Clearance properties of nano-sized particles and molecules as imaging agents: Considerations and caveats. Nanomedicine.

[31] Zhou Y., Dai Z. (2018). New strategies in the design of nanomedicines to oppose uptake by the mononuclear phagocyte system and enhance cancer therapeutic efficacy. Chem. Asian J..

[32] Huang Y., He S., Cao W., Cai K., Liang X.-J. (2012). Biomedical nanomaterials for imaging-guided cancer therapy. Nanoscale.

[33] Al-Jamal W., Al-Jamal K.T., Bomans P.H., Frederik P.M., Kostarelos K. (2008). Functionalized-quantum-dot-liposome hybrids as multimodal nanoparticles for cancer. Small.

[34] Soo Choi H., Liu W., Misra P., Tanaka E., Zimmer J.P., Itty Ipe B., Bawendi M.G., Frangioni J.V. (2007). Renal clearance of quantum dots. Nat. Biotechnol..

[35] Selim K.M.K., Ha Y.-S., Kim S.-J., Chang Y., Kim T.-J., Lee G.H., Kang I.-K. (2007). Surface modification of magnetite nanoparticles using lactobionic acid and their interaction with hepatocytes. Biomaterials.

[36] Park J.Y., Choi E.S., Baek M.J., Lee G.H., Woo S., Chang Y. (2009). Water-soluble Ultra Small paramagnetic or superparamagnetic metal oxide nanoparticles for molecular MR imaging. Eur. J. Inorg. Chem..

[37] McCarthy J.R., Weissleder R. (2008). Multifunctional magnetic nanoparticles for targeted imaging and therapy. Adv. Drug Deliv. Rev..

[38] Lee E.J., Heo W.C., Park J.W., Chang Y., Bae J.-E., Chae K.S., Kim T.J., Park J.A., Lee G.H. (2013). D-Glucuronic Acid Coated Gd(IO3)3·2H_2_O Nanomaterial as a Potential T1 MRI-CT Dual Contrast Agent. Eur. J. Inorg. Chem..

[39] Byrne J.D., Betancourt T., Brannon-Peppas L. (2008). Active targeting schemes for nanoparticle systems in cancer therapeutics. Adv. Drug Deliv. Rev..

[40] Patel D., Chang Y., Lee G.H. (2009). Amino acid functionalized magnetite nanoparticles in saline solution. Curr. Appl. Phys..

[41] Kim S.J., Xu W., Ahmad M.W., Baeck J.S., Chang Y., Bae J.E., Chae K.S., Kim T.J., Park J.A., Lee G.H. (2015). Synthesis of nanoparticle CT contrast agents: In vitro and in vivo studies. Sci. Technol. Adv. Mater..

[42] Kattel K., Park J.Y., Xu W., Bony B.A., Heo W.C., Tegafaw T., Kim C.R., Ahmad M.W., Jin S., Baeck J.S. (2013). Surface coated Eu(OH)^3^ nanorods: A facile synthesis, characterization, MR relaxivities and in vitro cytotoxicity. J. Nanosci. Nanotechnol..

[43] Popovtzer R., Agrawal A., Kotov N.A., Popovtzer A., Balter J., Carey T.E., Kopelman R. (2008). Targeted gold nanoparticles enable molecular CT imaging of cancer. Nano Lett..

[44] Ahmad M.W., Kim C.R., Baeck J.S., Chang Y., Kim T.J., Bae J.E., Chae K.S., Lee G.H. (2014). Bovine serum albumin (BSA) and cleaved-BSA conjugated ultrasmall Gd_2_O_3_ nanoparticles: Synthesis, characterization, and application to MRI contrast agents. Colloids Surf. A Physicochem. Eng. Asp..

[45] Tegafaw T., Xu W., Ahmad M.W., Xu M., Chang Y., Chae K.S., Kim T.J., Lee G.H. (2016). Fluorescent Brightener 28-Coated Fe3O4 Nanoparticles: Synthesis, Characterization, and Fluorescent Properties. J. Nanosci. Nanotechnol..

[46] Lartigue L., Coupeau M., Lesault M. (2020). Luminophore and magnetic multicore nanoassemblies for dual-mode MRI and fluorescence imaging. Nanomaterials.

[47] Krasia-Christoforou T., Socoliuc V., Knudsen K.D., Tombácz E., Turcu R., Vékás L. (2020). From single-core nanoparticles in ferrofluids to multi-core magnetic nanocomposites: Assembly strategies, structure, and magnetic behavior. Nanomaterials.

[48] Li J., Khalid A., Verma R., Abraham A., Qazi F., Dong X., Liang G., Tomljenovic-Hanic S. (2021). Silk fibroin coated magnesium oxide nanospheres: A biocompatible and biodegradable tool for noninvasive bioimaging applications. Nanomaterials.

[49] Mnasri W., Parvizian M., Ammar-Merah S. (2021). Design and Synthesis of Luminescent Lanthanide-Based Bimodal Nanoprobes for Dual Magnetic Resonance (MR) and Optical Imaging. Nanomaterials.

[50] Kharisov B.I., Dias H.V.R., Kharissova O.V., Vázquez A., Pena Y., Gomez I. (2014). Solubilization, dispersion and stabilization of magnetic nanoparticles in water and non-aqueous solvents: Recent trends. RSC Adv..

[51] Patel D., Moon J.Y., Chang Y., Kim T.J., Lee G.H. (2008). Poly(d,l-lactide-co-glycolide) coated superparamagnetic iron oxide nanoparticles: Synthesis, characterization and in vivo study as MRI contrast agent. Colloids Surf. A Physicochem. Eng. Asp..

[52] De Castro K.C., Costa J.M., Campos M.G.N. (2020). Drug-loaded polymeric nanoparticles: A review. Int. J. Polym. Mater. Polym. Biomater..

[53] Huang J., Wang L., Lin R., Wang A.Y., Yang L., Kuang M., Qian W., Mao H. (2013). Casein-coated iron oxide nanoparticles for high MRI contrast enhancement and efficient cell targeting. ACS Appl. Mater. Interfaces.

[54] Jurado R., Gálvez N. (2021). Apoferritin amyloid-fibril directed the in situ assembly and/or synthesis of optical and magnetic nanoparticles. Nanomaterials.

[55] Villaraza A.J.L., Bumb A., Brechbiel M.W. (2010). Macromolecules, dendrimers, and nanomaterials in magnetic resonance imaging: The interplay between size, function, and pharmacokinetics. Chem. Rev..

[56] Bellin M.-F. (2006). MR contrast agents, the old and the new. Eur. J. Radiol..

[57] Weinmann H.J., Brasch R.C., Press W.R., Wesbey G.E. (1984). Characteristics of gadolinium-DTPA complex: A potential NMR contrast agent. Am. J. Roentgenol..

[58] Rose T.A., Choi J.W. (2015). Intravenous imaging contrast media complications: The basics that every clinician needs to know. Am. J. Med..

[59] Chopra T., Kandukurti K., Shah S., Ahmed R., Panesar M. (2012). Understanding nephrogenic systemic fibrosis. Int. J. Nephrol..

[60] Marckmann P., Skov L., Rossen K., Dupont A., Damholt M.B., Heaf J.G., Thomsen H.S. (2006). Nephrogenic systemic fibrosis: Suspected causative role of gadodiamide used for contrast-enhanced magnetic resonance imaging. J. Am. Soc. Nephrol..

[61] Caravan P., Ellison J.J., McMurry T.J., Lauffer R.B. (1999). Gadolinium(III) chelates as MRI contrast agents: Structure, dynamics, and applications. Chem. Rev..

[62] Rowe M.D., Thamm D.H., Kraft S.L., Boyes S.G. (2009). Polymer-modified gadolinium metal-organic framework nanoparticles used as multifunctional nanomedicines for the targeted imaging and treatment of cancer. Biomacromolecules.

[63] Aime S., Botta M., Fasano M., Terreno E. (1998). Lanthanide (III) chelates for NMR biomedical applications. Chem. Soc. Rev..

[64] Aime S., Barge A., Cabella C., Crich S.G., Gianolio E. (2004). Targeting cells with MR imaging probes based on paramagnetic Gd (III) chelates. Curr. Pharm. Biotechnol..

[65] Aime S., Crich S.G., Gianolio E., Giovenzana G.B., Tei L., Terreno E. (2006). High sensitivity lanthanide (III) based probes for MR-medical imaging. Coord. Chem. Rev..

[66] Zhou Z., Huang D., Bao J., Chen Q., Liu G., Chen Z., Chen X., Gao J. (2012). A synergistically enhanced T1–T2 dual-modal contrast agent. Adv. Mater..

[67] Ahmad M.W., Xu W., Kim S.J., Baeck J.S., Chang Y., Bae J.E., Chae K.S., Park J.A., Kim T.J., Lee G.H. (2015). Potential dual imaging nanoparticle: Gd2O3 nanoparticle. Sci. Rep..

[68] Ananta J.S., Godin B., Sethi R., Moriggi L., Liu X., Serda R.E., Krishnamurthy R., Muthupillai R., Bolskar R.D., Helm L. (2010). Geometrical confinement of gadolinium-based contrast agents in nanoporous particles enhances T 1 contrast. Nat. Nanotechnol..

[69] Bridot J.-L., Faure A.-C., Laurent S., Rivière C., Billotey C., Hiba B., Janier M., Josserand V., Coll J.-L., Elst L.V. (2007). Hybrid gadolinium oxide nanoparticles: Multimodal contrast agents for in vivo imaging. J. Am. Chem. Soc..

[70] Park J.Y., Baek M.J., Choi E.S., Woo S., Kim J.H., Kim T.J., Jung J.C., Chae K.S., Chang Y., Lee G.H. (2009). Paramagnetic ultrasmall gadolinium oxide nanoparticles as advanced T1 MRI contrast agent: Account for large longitudinal relaxivity, optimal particle diameter, and in vivo T1 MR images. ACS Nano.

[71] Kim C.R., Baeck J.S., Chang Y., Bae J.E., Chae K.S., Lee G.H. (2014). Ligand-size dependent water proton relaxivities in ultrasmall gadolinium oxide nanoparticles and in vivo T1 MR images in a 1.5 T MR field. Phys. Chem. Chem. Phys..

[72] Tegafaw T., Xu W., Lee S.H., Chae K.S., Cha H., Chang Y., Lee G.H. (2016). Ligand-size and ligand-chain hydrophilicity effects on the relaxometric properties of ultrasmall Gd_2_O_3_ nanoparticles. AIP Adv..

[73] Bony B.A., Baeck J.S., Chang Y., Bae J.E., Chae K.S., Lee G.H. (2015). A Highly efficient new T1 MRI contrast agent with r2/r1 ≈ 1.0: Mixed Cu(II)/Gd(III) oxide nanoparticle. Bull. Korean Chem. Soc..

[74] Chang Y., Chae K.S., Lee G.H. (2016). Gadolinium agents for theragnosis of malignant tumors. Bioinspired Biomim. Nanobiomater..

[75] Ghaghada K.B., Starosolski Z.A., Bhayana S., Stupin I., Patel C.V., Bhavane R.C., Gao H., Bednov A., Yallampalli C., Belfort M. (2017). Pre-clinical evaluation of a nanoparticle-based blood-pool contrast agent for MR imaging of the placenta. Placenta.

[76] Rotz M.W., Culver K.S.B., Parigi G., MacRenaris K.W., Luchinat C., Odom T.W., Meade T.J. (2015). High relaxivity Gd (III)–DNA gold nanostars: Investigation of shape effects on proton relaxation. ACS Nano.

[77] Beija M., Li Y., Duong H.T., Laurent S., Vander Elst L., Muller R.N., Lowe A.B., Davis T.P., Boyer C. (2012). Polymer–gold nanohybrids with potential use in bimodal MRI/CT: Enhancing the relaxometric properties of Gd (III) complexes. J. Mater. Chem..

[78] Huang C.-L., Huang C.-C., Mai F.-D., Yen C.-L., Tzing S.-H., Hsieh H.-T., Ling Y.-C., Chang J.-Y. (2015). Application of paramagnetic graphene quantum dots as a platform for simultaneous dual-modality bioimaging and tumor-targeted drug delivery. J. Mater. Chem. B.

[79] Abou D.S., Thorek D.L.J., Ramos N.N., Pinkse M.W.H., Wolterbeek H.T., Carlin S.D., Beattie B.J., Lewis J.S. (2013). 89 Zr-labeled paramagnetic octreotide-liposomes for PET-MR imaging of cancer. Pharm. Res..

[80] Park J.-A., Kim J.Y., Lee Y.J., Lee W., Lim S.M., Kim T.-J., Yoo J., Chang Y., Kim K.M. (2013). Gadolinium complex of 125I/127I-RGD-DOTA conjugate as a tumor-targeting SPECT/MR bimodal imaging probe. ACS Med. Chem. Lett..

[81] Aryal M., Papademetriou I., Zhang Y.-Z., Power C., McDannold N., Porter T. (2019). MRI monitoring and quantification of ultrasound-mediated delivery of liposomes dually Labeled with gadolinium and fluorophore through the blood-brain barrier. Ultrasound Med. Biol..

[82] Hu D.-H., Sheng Z.-H., Zhang P.-F., Yang D.-Z., Liu S.-H., Gong P., Gao D.-Y., Fang S.-T., Ma Y.-F., Cai L.-T. (2013). Hybrid gold–gadolinium nanoclusters for tumor-targeted NIRF/CT/MRI triple-modal imaging in vivo. Nanoscale.

[83] Arifin D.R., Long C.M., Gilad A.A., Alric C., Roux S., Tillement O., Link T.W., Arepally A., Bulte J.W.M. (2011). Trimodal gadolinium-gold microcapsules containing pancreatic islet cells restore normoglycemia in diabetic mice and can be tracked by using US, CT, and positive-contrast MR imaging. Radiology.

[84] Wu B., Lu S.-T., Yu H., Liao R.-F., Li H., Zafitatsimo B.V.L., Li Y.-S., Zhang Y., Zhu X.-L., Liu H.-G. (2018). Gadolinium-chelate functionalized bismuth nanotheranostic agent for in vivo MRI/CT/PAI imaging-guided photothermal cancer therapy. Biomaterials.

[85] Hüber M.M., Staubli A.B., Kustedjo K., Gray M.H.B., Shih J., Fraser S.E., Jacobs R.E., Meade T.J. (1998). Fluorescently detectable magnetic resonance imaging agents. Bioconjug. Chem..

[86] Yan G.-P., Liu M.-L., Li L.-Y. (2005). Polyaspartamide gadolinium complexes containing sulfadiazine groups as potential macromolecular MRI contrast agents. Bioconjug. Chem..

[87] Bull S.R., Guler M.O., Bras R.E., Venkatasubramanian P.N., Stupp S.I., Meade T.J. (2005). Magnetic resonance imaging of self-assembled biomaterial scaffolds. Bioconjug. Chem..

[88] Bull S.R., Guler M.O., Bras R.E., Meade T.J., Stupp S.I. (2005). Self-assembled peptide amphiphile nanofibers conjugated to MRI contrast agents. Nano Lett..

[89] Anderson E.A., Isaacman S., Peabody D.S., Wang E.Y., Canary J.W., Kirshenbaum K. (2006). Viral nanoparticles donning a paramagnetic coat: Conjugation of MRI contrast agents to the MS2 capsid. Nano Lett..

[90] Langereis S., de Lussanet Q.G., van Genderen M.H.P., Backes W.H., Meijer E.W. (2004). Multivalent contrast agents based on gadolinium−diethylenetriaminepentaacetic acid-terminated poly (propylene imine) dendrimers for magnetic resonance imaging. Macromolecules.

[91] Talanov V.S., Regino C.A.S., Kobayashi H., Bernardo M., Choyke P.L., Brechbiel M.W. (2006). Dendrimer-based nanoprobe for dual modality magnetic resonance and fluorescence imaging. Nano Lett..

[92] Mulder W.J.M., Strijkers G.J., Griffioen A.W., van Bloois L., Molema G., Storm G., Koning G.A., Nicolay K. (2004). A liposomal system for contrast-enhanced magnetic resonance imaging of molecular targets. Bioconjug. Chem..

[93] Frias J.C., Williams K.J., Fisher E.A., Fayad Z.A. (2004). Recombinant HDL-like nanoparticles: A specific contrast agent for MRI of atherosclerotic plaques. J. Am. Chem. Soc..

[94] Accardo A., Tesauro D., Roscigno P., Gianolio E., Paduano L., D’Errico G., Pedone C., Morelli G. (2004). Physicochemical properties of mixed micellar aggregates containing CCK peptides and Gd complexes designed as tumor specific contrast agents in MRI. J. Am. Chem. Soc..

[95] Turner J.L., Pan D., Plummer R., Chen Z., Whittaker A.K., Wooley K.L. (2005). Synthesis of gadolinium-labeled shell-crosslinked nanoparticles for magnetic resonance imaging applications. Adv. Funct. Mater..

[96] Platas-Iglesias C., Vander Elst L., Zhou W., Muller R.N., Geraldes C.F.G.C., Maschmeyer T., Peters J.A. (2002). Zeolite GdNaY nanoparticles with very high relaxivity for application as contrast agents in magnetic resonance imaging. Chem. Eur. J..

[97] Bolskar R.D., Benedetto A.F., Husebo L.O., Price R.E., Jackson E.F., Wallace S., Wilson L.J., Alford J.M. (2003). First soluble M@ C60 derivatives provide enhanced access to metallofullerenes and permit in vivo evaluation of Gd@ C60 [C (COOH) 2] 10 as a MRI contrast agent. J. Am. Chem. Soc..

[98] Sitharaman B., Kissell K.R., Hartman K.B., Tran L.A., Baikalov A., Rusakova I., Sun Y., Khant H.A., Ludtke S.J., Chiu W. (2005). Superparamagnetic gadonanotubes are high-performance MRI contrast agents. Chem. Commun..

[99] Balkus K.J., Shi J. (1996). A study of suspending agents for gadolinium (III)-exchanged hectorite. An oral magnetic resonance imaging contrast agent. Langmuir.

[100] Lin Y.-S., Hung Y., Su J.-K., Lee R., Chang C., Lin M.-L., Mou C.-Y. (2004). Gadolinium (III)-incorporated nanosized mesoporous silica as potential magnetic resonance imaging contrast agents. J. Phys. Chem. B.

[101] Mulder W.J.M., Koole R., Brandwijk R.J., Storm G., Chin P.T.K., Strijkers G.J., de Mello Donegá C., Nicolay K., Griffioen A.W. (2006). Quantum dots with a paramagnetic coating as a bimodal molecular imaging probe. Nano Lett..

[102] Vuu K., Xie J., McDonald M.A., Bernardo M., Hunter F., Zhang Y., Li K., Bednarski M., Guccione S. (2005). Gadolinium-rhodamine nanoparticles for cell labeling and tracking via magnetic resonance and optical imaging. Bioconjug. Chem..

[103] Alric C., Serduc R., Mandon C., Taleb J., Le Duc G., Le Meur-Herland A., Billotey C., Perriat P., Roux S., Tillement O. (2008). Gold nanoparticles designed for combining dual modality imaging and radiotherapy. Gold Bull..

[104] Engström M., Klasson A., Pedersen H., Vahlberg C., Käll P.-O., Uvdal K. (2006). High proton relaxivity for gadolinium oxide nanoparticles. Magn. Reson. Mater. Phys. Biol. Med..

[105] McDonald M.A., Watkin K.L. (2003). Small particulate gadolinium oxide and gadolinium oxide albumin microspheres as multimodal contrast and therapeutic agents. Investig. Radiol..

[106] Roberts D., Zhu W.L., Frommen C.M., Rosenzweig Z. (2000). Synthesis of gadolinium oxide magnetoliposomes for magnetic resonance imaging. J. Appl. Phys..

[107] Evanics F., Diamente P.R., Van Veggel F., Stanisz G.J., Prosser R.S. (2006). Water-soluble GdF3 and GdF3/LaF3 nanoparticles physical characterization and NMR relaxation properties. Chem. Mater..

[108] Hu K.-W., Jhang F.-Y., Su C.-H., Yeh C.-S. (2009). Fabrication of Gd_2_O(CO_3_)2·H_2_O/silica/gold hybrid particles as a bifunctional agent for MR imaging and photothermal destruction of cancer cells. J. Mater. Chem..

[109] Li I., Su C., Sheu H., Chiu H., Lo Y., Lin W., Chen J., Yeh C. (2008). Gd_2_O (CO_3_)_2_H_2_O particles and the corresponding Gd_2_O_3_: Synthesis and applications of magnetic resonance contrast agents and template particles for hollow spheres and hybrid composites. Adv. Funct. Mater..

[110] Nwe K., Milenic D., Bryant L.H., Regino C.A.S., Brechbiel M.W. (2011). Preparation, characterization and in vivo assessment of Gd-albumin and Gd-dendrimer conjugates as intravascular contrast-enhancing agents for MRI. J. Inorg. Biochem..

[111] Ma X.-H., Gong A., Xiang L.-C., Chen T.-X., Gao Y.-X., Liang X.-J., Shen Z.-Y., Wu A.-G. (2013). Biocompatible composite nanoparticles with large longitudinal relaxivity for targeted imaging and early diagnosis of cancer. J. Mater. Chem. B.

[112] Ahmad M.Y., Cha H., Oh I.-T., Tegafaw T., Miao X., Ho S.L., Marasini S., Ghazanfari A., Yue H., Chae K.S. (2018). Synthesis, characterization, and enhanced cancer-imaging application of trans-activator of transcription peptide-conjugated ultrasmall gadolinium oxide nanoparticles. Bull. Korean Chem. Soc..

[113] Park J., Lee J., Jung J., Yu D., Oh C., Ha S., Kim T., Chang Y. (2008). Gd-DOTA conjugate of RGD as a potential tumor-targeting MRI contrast agent. ChemBioChem.

[114] Yang Z., He W., Zheng H., Wei J., Liu P., Zhu W., Lin L., Zhang L., Yi C., Xu Z. (2018). One-pot synthesis of albumin-gadolinium stabilized polypyrrole nanotheranostic agent for magnetic resonance imaging guided photothermal therapy. Biomaterials.

[115] Bertozzi C.R., Kiessling L.L. (2001). Chemical glycobiology. Science.

[116] Martinelli J., Fekete M., Tei L., Botta M. (2011). Cleavable β-cyclodextrin nanocapsules incorporating Gd III-chelates as bioresponsive MRI probes. Chem. Commun..

[117] Gambino G., Engelmann J., Tei L., Botta M., Logothetis N.K., Mamedov I. (2013). Multimodal contrast agents for in vivo neuroanatomical analysis of monosynaptic connections. Biomaterials.

[118] Zhang W., Chen Y., Huang Z.W., Cai L., He L. (2011). The synthesis of a D-glucosamine contrast agent, Gd-DTPA-DG, and its application in cancer molecular imaging with MRI. Eur. J. Radiol..

[119] Termsarasab U., Cho H.-J., Moon H.T., Park J.-H., Yoon I.-S., Kim D.-D. (2013). Self-assembled magnetic resonance imaging nanoprobes based on arachidyl chitosan for cancer diagnosis. Colloids Surf. B Biointerfaces.

[120] Mortezazadeh T., Gholibegloo E., Alam N.R., Dehghani S., Haghgoo S., Ghanaati H., Khoobi M. (2019). Gadolinium (III) oxide nanoparticles coated with folic acid-functionalized poly (β-cyclodextrin-co-pentetic acid) as a biocompatible targeted nano-contrast agent for cancer diagnostic: In vitro and in vivo studies. Magn. Reson. Mater. Phys. Biol. Med..

[121] Bony B.A., Baeck J.S., Chang Y., Bae J.E., Chae K.S., Lee G.H. (2014). Water-soluble D-glucuronic acid coated ultrasmall mixed Ln/Mn (Ln = Gd and Dy) oxide nanoparticles and their application to magnetic resonance imaging. Biomater. Sci..

[122] Xu W., Miao X., Oh I., Chae K.S., Cha H., Chang Y., Lee G.H. (2016). Dextran-coated ultrasmall Gd_2_O_3_ nanoparticles as potential T1 MRI contrast agent. ChemistrySelect.

[123] Hifumi H., Yamaoka S., Tanimoto A., Akatsu T., Shindo Y., Honda A., Citterio D., Oka K., Kuribayashi S., Suzuki K. (2009). Dextran coated gadolinium phosphate nanoparticles for magnetic resonance tumor imaging. J. Mater. Chem..

[124] Ahmad M.Y., Ahmad M., Yue H., Ho S.L., Cha H., Marasini S., Tegafaw T., Liu S., Ghazanfari A., Chae K.-S. (2021). Chitosan oligosaccharide lactate-coated ultrasmall gadolinium oxide nanoparticles: Synthesis, in vitro cytotoxicity, and relaxometric properties. J. Nanosci. Nanotechnol..

[125] Cheng Z., Al Zaki A., Jones I.W., Hall H.K., Aspinwall C.A., Tsourkas A. (2014). Stabilized porous liposomes with encapsulated Gd-labeled dextran as a highly efficient MRI contrast agent. Chem. Commun..

[126] Hossann M., Wang T., Syunyaeva Z., Wiggenhorn M., Zengerle A., Issels R.D., Reiser M., Lindner L.H., Peller M. (2013). Non-ionic Gd-based MRI contrast agents are optimal for encapsulation into phosphatidyldiglycerol-based thermosensitive liposomes. J. Control. Release.

[127] Nitta N., Takakusagi Y., Kokuryo D., Shibata S., Tomita A., Higashi T., Aoki I., Harada M. (2018). Intratumoral evaluation of 3D microvasculature and nanoparticle distribution using a gadolinium-dendron modified nano-liposomal contrast agent with magnetic resonance micro-imaging. Nanomed. Nanotechnol. Biol. Med..

[128] Machová E., O’Regan S., Newcombe J., Meunier F., Prentice J., Dove R., Lisá V., Doležal V. (2009). Detection of choline transporter-like 1 protein CTL1 in neuroblastoma× glioma cells and in the CNS, and its role in choline uptake. J. Neurochem..

[129] Lattuada L., Lux G. (2003). Synthesis of Gd-DTPA-cholesterol: A new lipophilic gadolinium complex as a potential MRI contrast agent. Tetrahedron Lett..

[130] Rui M., Guo W., Ding Q., Wei X., Xu J., Xu Y. (2012). Recombinant high-density lipoprotein nanoparticles containing gadolinium-labeled cholesterol for morphologic and functional magnetic resonance imaging of the liver. Int. J. Nanomed..

[131] Ye F., Ke T., Jeong E.-K., Wang X., Sun Y., Johnson M., Lu Z.-R. (2006). Noninvasive visualization of in vivo drug delivery of poly (L-glutamic acid) using contrast-enhanced MRI. Mol. Pharm..

[132] Shiraishi K., Kawano K., Maitani Y., Yokoyama M. (2010). Polyion complex micelle MRI contrast agents from poly (ethylene glycol)-b-poly (L-lysine) block copolymers having Gd-DOTA; preparations and their control of T1-relaxivities and blood circulation characteristics. J. Control. Release.

[133] Liu Q., Zhu H., Qin J., Dong H., Du J. (2014). Theranostic vesicles based on bovine serum albumin and poly (ethylene glycol)-block-poly (L-lactic-co-glycolic acid) for magnetic resonance imaging and anticancer drug delivery. Biomacromolecules.

[134] Liu Y., Chen Z., Liu C., Yu D., Lu Z., Zhang N. (2011). Gadolinium-loaded polymeric nanoparticles modified with Anti-VEGF as multifunctional MRI contrast agents for the diagnosis of liver cancer. Biomaterials.

[135] Ho S.L., Cha H., Oh I.T., Jung K.-H., Kim M.H., Lee Y.J., Miao X., Tegafaw T., Ahmad M.Y., Chae K.S. (2018). Magnetic resonance imaging, gadolinium neutron capture therapy, and tumor cell detection using ultrasmall Gd_2_O_3_ nanoparticles coated with polyacrylic acid-rhodamine B as a multifunctional tumor theragnostic agent. RSC Adv..

[136] Jang Y.-J., Liu S., Yue H., Park J., Cha H., Ho S.L., Marasini S., Ghazanfari A., Ahmad M.Y., Miao X. (2021). Hydrophilic biocompatible poly (acrylic acid-co-maleic acid) polymer as a surface-coating ligand of ultrasmall Gd2O3 nanoparticles to obtain a high r1 value and T1 MR images. Diagnostics.

[137] Ahmad M.Y., Ahmad M.W., Yue H., Ho S.L., Park J.A., Jung K.-H., Cha H., Marasini S., Ghazanfari A., Liu S. (2020). In vivo positive magnetic resonance imaging applications of poly(methyl vinyl ether-alt-maleic acid)-coated ultra-small paramagnetic gadolinium oxide nanoparticles. Molecules.

[138] Yon M., Gineste S., Parigi G., Lonetti B., Gibot L., Talham D.R., Marty J.-D., Mingotaud C. (2021). Hybrid Polymeric Nanostructures Stabilized by Zirconium and Gadolinium Ions for Use as Magnetic Resonance Imaging Contrast Agents. ACS Appl. Nano Mater..

[139] Hu X., Tang Y., Hu Y., Lu F., Lu X., Wang Y., Li J., Li Y., Ji Y., Wang W. (2019). Gadolinium-chelated conjugated polymer-based nanotheranostics for photoacoustic/magnetic resonance/NIR-II fluorescence imaging-guided cancer photothermal therapy. Theranostics.

[140] Grogna M., Cloots R., Luxen A., Jérôme C., Desreux J.-F., Detrembleur C. (2011). Design and synthesis of novel DOTA (Gd 3+)–polymer conjugates as potential MRI contrast agents. J. Mater. Chem..

[141] Kang M.-K., Lee G.H., Jung K.-H., Jung J.-C., Kim H.-K., Kim Y.-H., Lee J., Ryeom H.-K., Kim T.-J., Chang Y. (2016). Gadolinium Nanoparticles Conjugated with Therapeutic Bifunctional Chelate as a Potential T1 Theranostic Magnetic Resonance Imaging Agent. J. Biomed. Nanotechnol..

[142] Kim H.-K., Kang M.-K., Jung K.-H., Kang S.-H., Kim Y.-H., Jung J.-C., Lee G.H., Chang Y., Kim T.-J. (2013). Gadolinium complex of DO3A-benzothiazole aniline (BTA) conjugate as a theranostic agent. J. Med. Chem..

[143] Yue H., Marasini S., Ahmad M.Y., Ho S.L., Cha H., Liu S., Jang Y.J., Tegafaw T., Ghazanfari A., Miao X. (2020). Carbon-coated ultrasmall gadolinium oxide (Gd2O3@C) nanoparticles: Application to magnetic resonance imaging and fluorescence properties resonance imaging and fluorescence properties. Colloids Surfaces A Physicochem. Eng. Asp..

[144] Wang F., Peng E., Zheng B., Li S.F.Y., Xue J.M. (2015). Synthesis of water-dispersible Gd2O3/GO nanocomposites with enhanced MRI T 1 relaxivity. J. Phys. Chem. C.

[145] Lee B.H., Hasan M.T., Lichthardt D., Gonzalez-Rodriguez R., Naumov A. (2020). V Manganese–nitrogen and gadolinium–nitrogen Co-doped graphene quantum dots as bimodal magnetic resonance and fluorescence imaging nanoprobes. Nanotechnology.

[146] Li Y., Dong H., Tao Q., Ye C., Yu M., Li J., Zhou H., Yang S., Ding G., Xie X. (2020). Enhancing the magnetic relaxivity of MRI contrast agents via the localized superacid microenvironment of graphene quantum dots. Biomaterials.

[147] Carniato F., Muñoz-Úbeda M., Tei L., Botta M. (2015). Selective functionalization of mesoporous silica nanoparticles with ibuprofen and Gd (III) chelates: A new probe for potential theranostic applications. Dalton Trans..

[148] He K., Li J., Shen Y., Yu Y. (2019). pH-Responsive polyelectrolyte coated gadolinium oxide-doped mesoporous silica nanoparticles (Gd2O3@MSNs) for synergistic drug delivery and magnetic resonance imaging enhancement. J. Mater. Chem. B.

[149] Liu J., Xiong Z., Zhang J., Peng C., Klajnert-Maculewicz B., Shen M., Shi X. (2019). Zwitterionic gadolinium (III)-complexed dendrimer-entrapped gold nanoparticles for enhanced computed tomography/magnetic resonance imaging of lung cancer metastasis. ACS Appl. Mater. Interfaces.

[150] Tegafaw T., Xu W., Ahmad M.W., Baeck J.S., Chang Y., Bae J.E., Chae K.S., Kim T.J., Lee G.H. (2015). Dual-mode T1 and T2 magnetic resonance imaging contrast agent based on ultrasmall mixed gadolinium-dysprosium oxide nanoparticles: Synthesis, characterization, and in vivo application. Nanotechnology.

[151] Choi E.S., Park J.Y., Baek M.J., Xu W., Kattel K., Kim J.H., Lee J.J., Chang Y., Kim T.J., Bae J.E. (2010). Water-soluble ultra-small manganese oxide surface doped gadolinium oxide (Gd_2_O_3_@MnO) nanoparticles for MRI contrast agent. Eur. J. Inorg. Chem..

[152] Tegafaw T., Bony B.A., Xu W., Cha H., Chang Y., Lee S.-H., Chae K.S., Lee G.H. (2017). Longitudinal water proton relaxivity and in vivo T 1 MR images of mixed Zn(II)/Gd(III) oxide nanoparticles. J. Nanosci. Nanotechnol..

[153] Xu W., Park J.Y., Kattel K., Bony B.A., Heo W.C., Jin S., Park J.W., Chang Y., Do J.Y., Chae K.S. (2012). A T1, T2 magnetic resonance imaging (MRI)-fluorescent imaging (FI) by using ultrasmall mixed gadolinium–europium oxide nanoparticles. New J. Chem..

[154] Kim H.-K., Lee J.-J., Choi G., Sung B., Kim Y.-H., Baek A.R., Kim S., Song H., Kim M., Cho A.E. (2020). Gadolinium-based neuroprognostic magnetic resonance imaging agents suppress COX-2 for prevention of reperfusion injury after stroke. J. Med. Chem..

[155] Choi G., Kim H.-K., Baek A.R., Kim S., Kim M.J., Kim M., Cho A.E., Lee G.-H., Jung H., Yang J.-U. (2020). Multifunctional imaging of amyloid-beta peptides with a new gadolinium-based contrast agent in Alzheimer’s disease. J. Ind. Eng. Chem..

[156] Park J.Y., Kim S.J., Lee G.H., Jin S., Chang Y., Bae J.E., Chae K.S. (2015). Various ligand-coated ultrasmall gadolinium-oxide nanoparticles: Water proton relaxivity and in-vivo T1 MR image. J. Korean Phys. Soc..

[157] Baek A.R., Kim H.-K., Park S., Lee G.H., Kang H.J., Jung J.-C., Park J.-S., Ryeom H.-K., Kim T.-J., Chang Y. (2017). Gadolinium complex of 1,4,7,10-tetraazacyclododecane-1,4,7-trisacetic acid (DO3A)-ethoxybenzyl (EOB) conjugate as a new macrocyclic hepatobiliary MRI contrast agent. J. Med. Chem..

[158] Miao X., Xu W., Cha H., Chang Y., Oh I.T., Chae K.S., Lee G.H. (2017). Application of dye-coated ultrasmall gadolinium oxide nanoparticles for biomedical dual imaging. Bull. Korean Chem. Soc..

[159] Xu W., Park J.Y., Kattel K., Ahmad M.W., Bony B.A., Heo W.C., Jin S., Park J.W., Chang Y., Kim T.J. (2012). Fluorescein-polyethyleneimine coated gadolinium oxide nanoparticles as T1 magnetic resonance imaging (MRI)–cell labeling (CL) dual agents. RSC Adv..

[160] Jung K.-H., Kim H.-K., Lee G.H., Kang D.-S., Park J.-A., Kim K.M., Chang Y., Kim T.-J. (2011). Gd complexes of macrocyclic diethylenetriaminepentaacetic acid (DTPA) biphenyl-2,2′-bisamides as strong blood-pool magnetic resonance imaging contrast agents. J. Med. Chem..

[161] Gu S., Kim H.-K., Lee G.H., Kang B.-S., Chang Y., Kim T.-J. (2011). Gd-complexes of 1,4,7,10-tetraazacyclododecane- N, N′, N″, N‴-1,4,7,10-tetraacetic acid (DOTA) conjugates of tranexamates as a new class of blood-pool magnetic resonance imaging contrast agents. J. Med. Chem..

